# Increased experimental conditions and marker densities identified more genetic loci associated with southern and northern leaf blight resistance in maize

**DOI:** 10.1038/s41598-018-25304-z

**Published:** 2018-05-01

**Authors:** Yong-xiang Li, Lin Chen, Chunhui Li, Peter J. Bradbury, Yun-su Shi, Yanchun Song, Dengfeng Zhang, Zhiwu Zhang, Edward S. Buckler, Yu Li, Tianyu Wang

**Affiliations:** 1 Institute of Crop Sciences, Chinese Academy of Agricultural Sciences, Beijing, 100081 China; 2 Institute for Genomic Diversity, Cornell University, Ithaca, NY 14853 USA; 3 United States Department of Agriculture-Agricultural Research Service, Ithaca, NY 14853 USA; 4 Department of Crop and Soil Sciences, Washington State University, Pullman, WA 99164 USA

## Abstract

Southern leaf blight (SLB) and northern leaf blight (NLB) are the two major foliar diseases limiting maize production worldwide. Upon previous study with the nested association mapping (NAM) population, which consist of 5,000 recombinant inbred lines from 25 parents crossed with B73, we expanded the phenotyping environments from the United States (US) to China, and increased the marker densities from 1106 to 7386 SNPs for linkage mapping, and from 1.6 to 28.5 million markers for association mapping. We identified 49 SLB and 48 NLB resistance-related unique QTLs in linkage mapping, and multiple loci in association mapping with candidate genes involved in known plant disease-resistance pathways. Furthermore, an independent natural population with 282 diversified inbred lines were sequenced for four candidate genes selected based on their biological functions. Three of them demonstrated significant associations with disease resistance. These findings provided valuable resources for further implementations to develop varieties with superior resistance for NLB and SLB.

## Introduction

Southern leaf blight (SLB), caused by the fungus *Cochliobolus heterostrophus*, and northern leaf blight (NLB), caused by the fungus *Exserohilum turcicum*, are two major foliar diseases that can lead to serious yield loss in maize-growing regions worldwide^1,2^. However, there are no genes causing complete immunity to these two foliar diseases^3–6^. Therefore, quantitative disease resistance (QDR) has been widely used to oppose these two diseases in maize breeding programmes^7–9^.

Plants have evolved both complete resistance, conditioned by a single gene (qualitative resistance), and incomplete resistance, conditioned by multiple genes of partial effect (quantitative resistance)^10,11^. Genes associated with qualitative resistance, often termed R-genes, are usually race specific and generally confer a high level of resistance^12^. Among the six classes of R-genes, the most common class contains characteristic nucleotide binding-leucine rich repeats (NB-LRRs or NLRs)^10,13^. R-genes are important in plant disease resistance systems. However, the lack of durability (failure of pathogen recognition) and availability (particularly for necrotrophic systems) of R-genes has limited their application in crop protection^10^. In contrast, QDR in plants is of practical importance in agriculture because it is less readily overcome by the evolution of pathogens than simply inherited forms of resistance^14^. However, many quantitative resistance loci (QRLs), such as the NLB resistance-related genes *Ht*^15,16^, *Ht*2^17–19^ and *Htn1*^20^, have been demonstrated to be race specific. Co-localization of R-genes and QRLs has been observed in several crops, including rice^21^, potato^22^, and maize^15^. QRLs have been assumed to simply be weaker forms of R-genes^23^. Therefore, a better understanding of the relationship between R-genes and QRLs would contribute to crop disease management, particularly for diseases caused by necrotrophic pathogens.

Both SLB and NLB are caused by necrotrophic pathogens; these pathogens depend on killing host cells and then living in the dead tissue. Resistance to these pathogens is mostly conferred in quantitative manner and can present additive or incomplete effects^16–20,24^. Recently, considerable progress has been made concerning the genetic dissection of SLB and NLB resistance. For example, using a nested association mapping (NAM) population with 5000 RILs obtained from 25 crosses with a common parent (B73)^25^, the genetic architecture of SLB^26^ and NLB resistance^27^ has been analysed. The results demonstrated that resistance of SLB and NLB is predominantly determined by numerous loci with small additive effects. Through a genome-wide association study (GWAS) of the maize NAM population, multiple candidate genes involved in basal defence against SLB and NLB were identified, which suggested that QDR in plants is conditioned by a range of mechanisms, including basal resistance^26,27^. Moreover, a wall-associated receptor-like kinase gene, *ZmWAKRLK1* (*Htn1*)^4^, and one remorin gene, *ZmREM6.3*^6^, were shown to be involved in NLB resistance. Recently, a gene encoding maize caffeoyl-CoA O-methyltransferase (*ZmCCoAOMT*2) was identified to confer quantitative resistance to multiple foliar maize diseases, including the resistance to SLB^28^.

There are two aspects to further enhance our understanding the resistance mechanisms against SLB and NLB through characterization of resistance-implicated genes among known SLB and NLB R-genes, NB-LRR-type genes^29,30^ and other disease resistance homologues. One is the dependence of their phenotypic performance to environments. Expansion of testing environments may benefits the dissection of resistance genetic architecture. The other is the gap between academic research and breeding practice. Research is usually conducted with artificial inoculation, however, breeding aims to the resistance under natural condition. Previously, the SLB and NLB resistance of an NAM population was mainly studied under the artificially inoculated conditions present in North America^26,27^.

In this study, the SLB and NLB resistance of this NAM population was evaluated under naturally inoculated conditions in China. The objective of the present study was to conduct genetic dissection of SLB and NLB resistance with increased environments and marker density to further elucidate the genetic bases of QDR.

## Results

### Consistency of SLB and NLB resistance across environments

**Figure 1:**
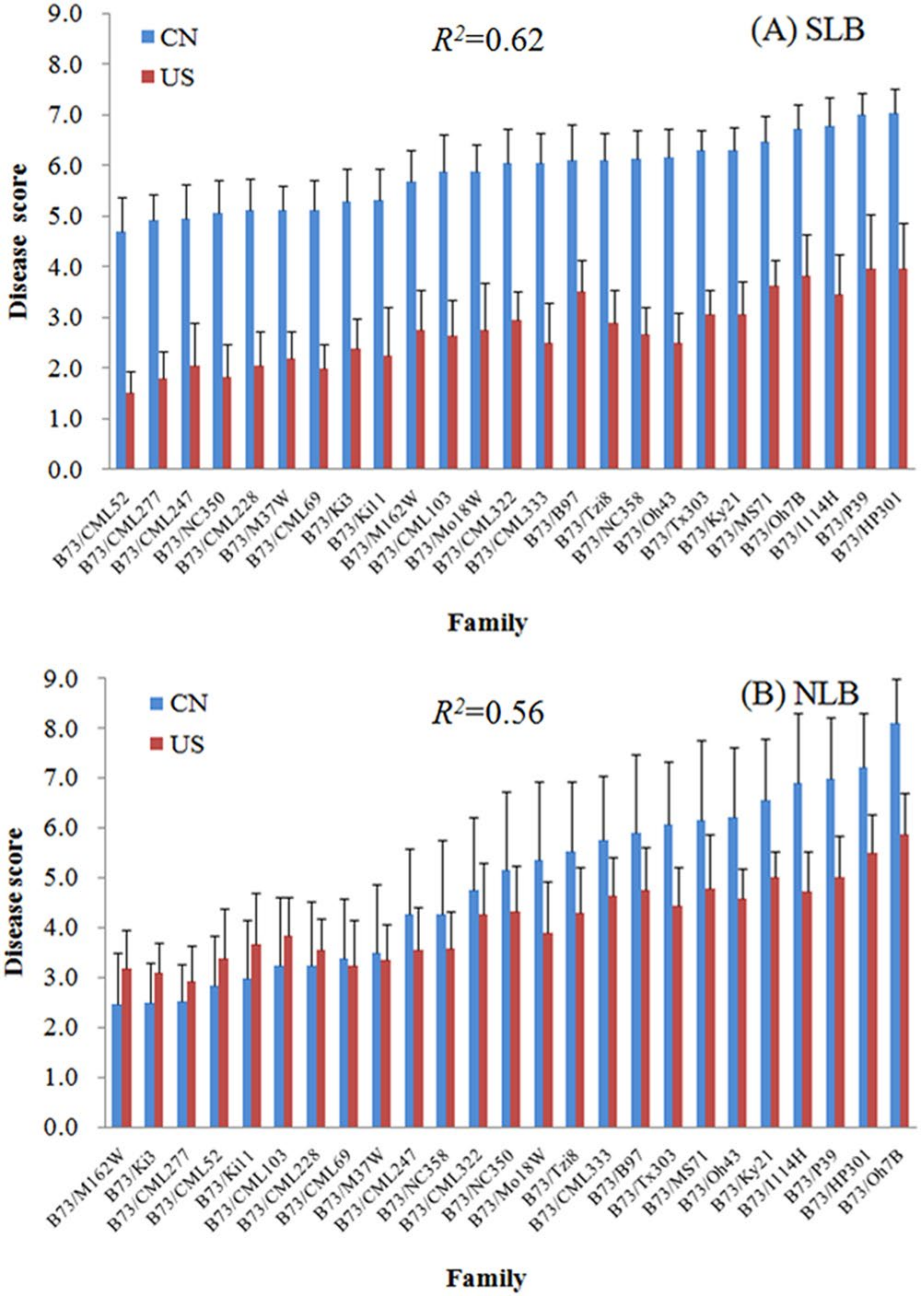
RIL family mean +St. Dev. of southern leaf blight (SLB) and northern leaf blight (NLB) disease scores in the United States (US) and China (CN). (**A**) SLB, and (**B**) NLB. Blue bars, the disease scores for the naturally inoculated conditions in China; red bars, the best linear unbiased predictions (BLUPs) of the disease scores in the US environments. The correlations (*R*^*2*^) were calculated using a linear model for all NAM RILs between the disease performance in the US and China.

A maize NAM population composed of 5,000 recombinant inbred lines (RILs) derived from crosses of B73 as common parent with other 25 diverse parent inbred lines^25^, was used for this study. Among the 5,000 RILs, 4694 RILs were scored for the SLB under one naturally inoculated environment and two artificially inoculated environments in the United States (US)^26^. Best Linear Unbiased Predictions (BLUPs) were derived for 4413 RILs by using a mixed model^31^. Similarly, the NLB resistance of 4042 RILs from this NAM population were scored under three artificially inoculated environments in the US, and the corresponding BLUPs were derived^27^. We scored 4211 RILs from this NAM population under naturally inoculated environments in China (three environments for SLB and one environment for NLB) (Supplementary Dataset S1). To eliminate environmental influences, the BLUPs of the SLB scores were calculated in SAS using PROC MIXED (Version 9.2; SAS Institute, Cary, NC, USA), with the genotype and environment as the random effects^32^. Strong correlations between the US and China were observed for SLB (*R*^2^ = 0.62) and NLB (*R*^2^ = 0.56) (Fig. 1).

About 10% of the NAM RILs were scored in China under artificially inoculated environment with the pathogen responsible for SLB (*C. heterostrophus* race O) or NLB (*E. turcicum* race 1) (Supplementary Dataset S1). These RILS include 488 RILs for SLB and 529 RILs for NLB. Strong correlations were observed between the artificially and naturally inoculated environments for both SLB (*R*^2^ = 0.79) and NLB (*R*^2^ = 0.67).

Generally, necrotrophic leaf diseases, including SLB and NLB, tend to occur before anthesis and the symptoms become much more severe after anthesis^12^. For the NAM population used this study, the days to anthesis (DTA) have been recorded under 13 environments, and the BLUPs of the DTA for the NAM RILs have been calculated^33^. The correlations among SLB, NLB and DTA were displayed using a contour plot (Supplementary Fig. S1). The NAM RILs with a later flowering time generally showed higher disease resistance, and certain correlations were observed between the disease scores and DTA (SLB: *r* = −0.56*, p* < 6.5 × 10^−322^ in China, *r* = −0.52*, p* < 3.1 × 10^−300^ in the US; NLB: *r* = −0.47*, p* < 2.1 × 10^−205^ in China, *r* = −0.38*, p* < 5.3 × 10^−143^ in the US). We also observed certain phenotypic correlations between SLB and NLB scored in the US (*R*^2^ = 0.25) and China (*R*^2^ = 0.35).

### Identification of SLB and NLB resistance QTLs

Based on the genotypes obtained using the Genotyping By Sequencing (GBS) approach^34^, a consensus genetic map including 7386 single-nucleotide polymorphisms (SNPs) (with a uniform genetic distance of 0.2 cM) was constructed for the NAM population, which has been proven to be a more powerful approach for QTL detection than using a linkage map of 1106 SNPs^31^. Employing the 7386-SNP linkage map, we conducted QTL mapping via the joint linkage analysis by using stepwise regression method^35^ (Table 1; Supplementary Table S1). Using the US disease scores, we identified 37 and 40 QTLs for SLB and NLB resistance, respectively, explaining 86% and 85% of the total phenotypic variance. Using the phenotypes collected in China, we identified 27 and 16 QTLs for SLB and NLB, respectively. More than half of the QTLs were overlapped between using the US and Chinese scores (15 QTLs for SLB, 8 QTLs for NLB). The QTLs identified under China environments, including the overlapped ones, explained 81% and 75% of the phenotypic variance in the US resistance performance for SLB and NLB, respectively.

**Table 1:** QTLs identified by joint linkage mapping of SLB and NLB resistance.

Trait	US		China		Overlapped QTLs between the environments of the US and China
	QTL Number	Phenotypic variance explained (%)	QTL Number	Phenotypic variance explained (%)	
SLB	37	86	27	81	15
NLB	40	85	16	75	8

SLB, southern leaf blight; NLB, northern leaf blight; US, the United States.

**Figure 2:**
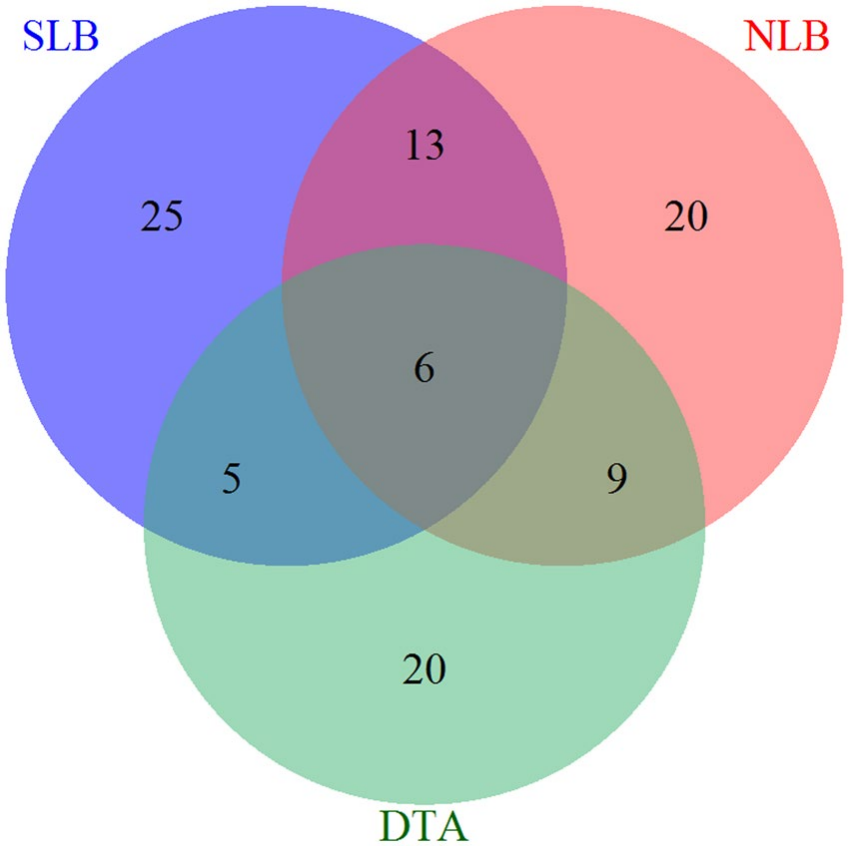
Number of quantitative trait loci (QTLs) shared among SLB and NLB resistance and the days to anthesis (DTA).

Combining the mapping results of the US and China, 49 for SLB and 48 for NLB resistance-related unique QTLs were identified (Supplementary Table S2). A total of 40 DTA QTLs were also identified using the BULPs across the multiple environments (Supplementary Table S1). The resistance-related unique QTLs contained many pleiotropic loci affecting both disease resistance and flowering time (Fig. 2). Importantly, 19 unique SLB QTLs overlapped with unique NLB QTLs, and 11 unique QTLs for SLB and 15 unique QTLs for NLB overlapped with the confidence intervals of DTA QTLs. Moreover, six regions associated with all three traits examined were identified (Supplementary Table S2).

### Co-localization test between resistance-implicated genes and identified QTLs

**Figure 3:**
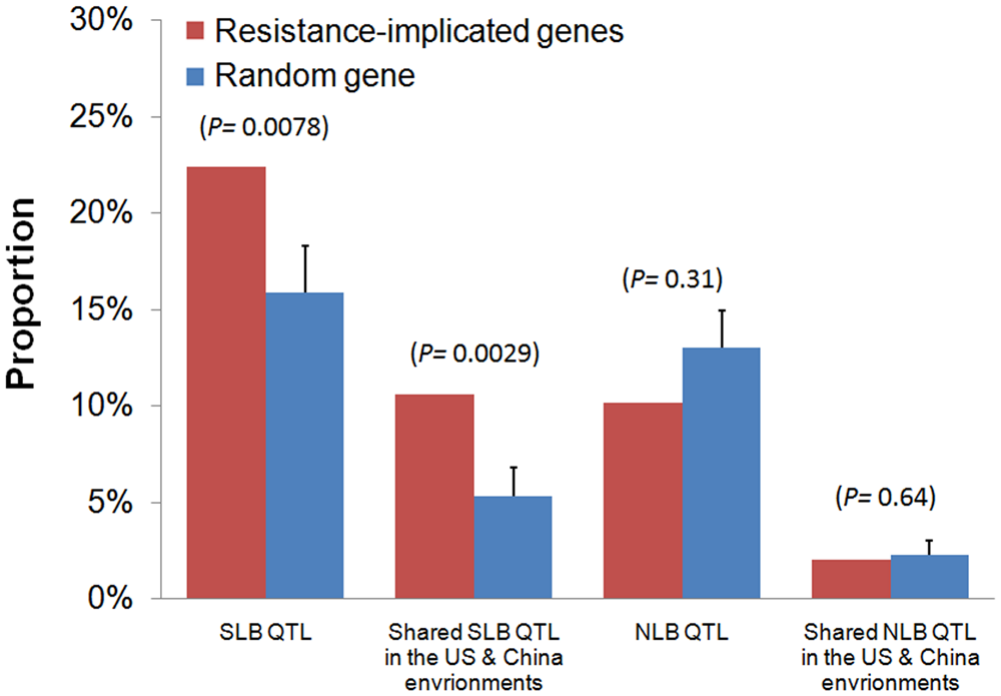
Co-localization test between resistance-implicated genes and SLB and NLB resistance-related unique QTLs. Co-localization is demonstrated as the difference between the observed (red bars) and expected (blue bars) proportions of resistance-implicated genes within QTL regions for SLB or NLB resistance. The expected proportion was derived from a number of randomly selected genes equal to the number of the observed resistance-implicated genes. The random process was replicated 1000 times. The standard errors were used for statistical tests and are displayed for the corresponding expectations.

Based on a literature search, a set of 245 resistance-implicated genes (Supplementary Dataset S2), including NB-LRR type genes from previous publications^29,30^, the identified SLB and NLB R-genes^4,6,28^ and disease resistance homologues in MaizeGDB (http://www.maizegdb.org/) determined through genome sequence analysis, were selected to conduct the co-localization test. We observed 54 and 25 resistance-implicated genes within the SLB and NLB resistance QTLs, respectively. Significant co-localization was observed for SLB (*P* = 0.0078), and not for NLB, resistance-related unique QTLs (Fig. 3). We identified four SLB resistance QTLs clustering with at least five resistance-implicated genes (mainly NB-LRR genes) in each region (Supplementary Fig. S2). Two of these QTLs were located in bins 4.01 (12 resistance-implicated genes, i.e., GRMZM2G043137, GRMZM2G053817, GRMZM2G455909, GRMZM2G155305, GRMZM2G091726, GRMZM2G308369, GRMZM2G087974, GRMZM2G013170, GRMZM2G311664, GRMZM2G455321, GRMZM2G156346, GRMZM2G128241) and 4.08 (5 resistance-implicated genes, i.e., GRMZM2G549240, GRMZM2G158316, GRMZM2G380784, GRMZM5G880361, GRMZM2G050959) and the other two were located in bins 6.01 (8 resistance-implicated genes, i.e., GRMZM2G054946, AC193598.3_FGP002, GRMZM2G364977, GRMZM2G306727, GRMZM2G020980, AC205909.3_FGP001, GRMZM2G334584, GRMZM2G136662) and 10.01 (15 resistance-implicated genes, i.e., GRMZM2G004412, GRMZM2G060884, AC152495.1_FGP002, AC152495.1_FGP003, AC152495.1_FGP010, AC152495.1_FGP015, AC152495.1_FGP017, GRMZM5G879178, GRMZM2G069382, GRMZM2G083258, GRMZM2G143769, GRMZM2G443939, GRMZM2G003625, GRMZM2G061742, GRMZM2G005134). These co-localized resistance-implicated genes within QTLs might be important for SLB resistance. We also observed six resistance-implicated genes falling within the common resistance regions for SLB and NLB (GRMZM2G438824, GRMZM2G017629, GRMZM2G318882 on chromosome 1; GRMZM5G856249 on chromosome 2; and GRMZM2G032751 and GRMZM2G142680 on chromosome 10).

### GWAS of SLB and NLB resistance

**Figure 4:**
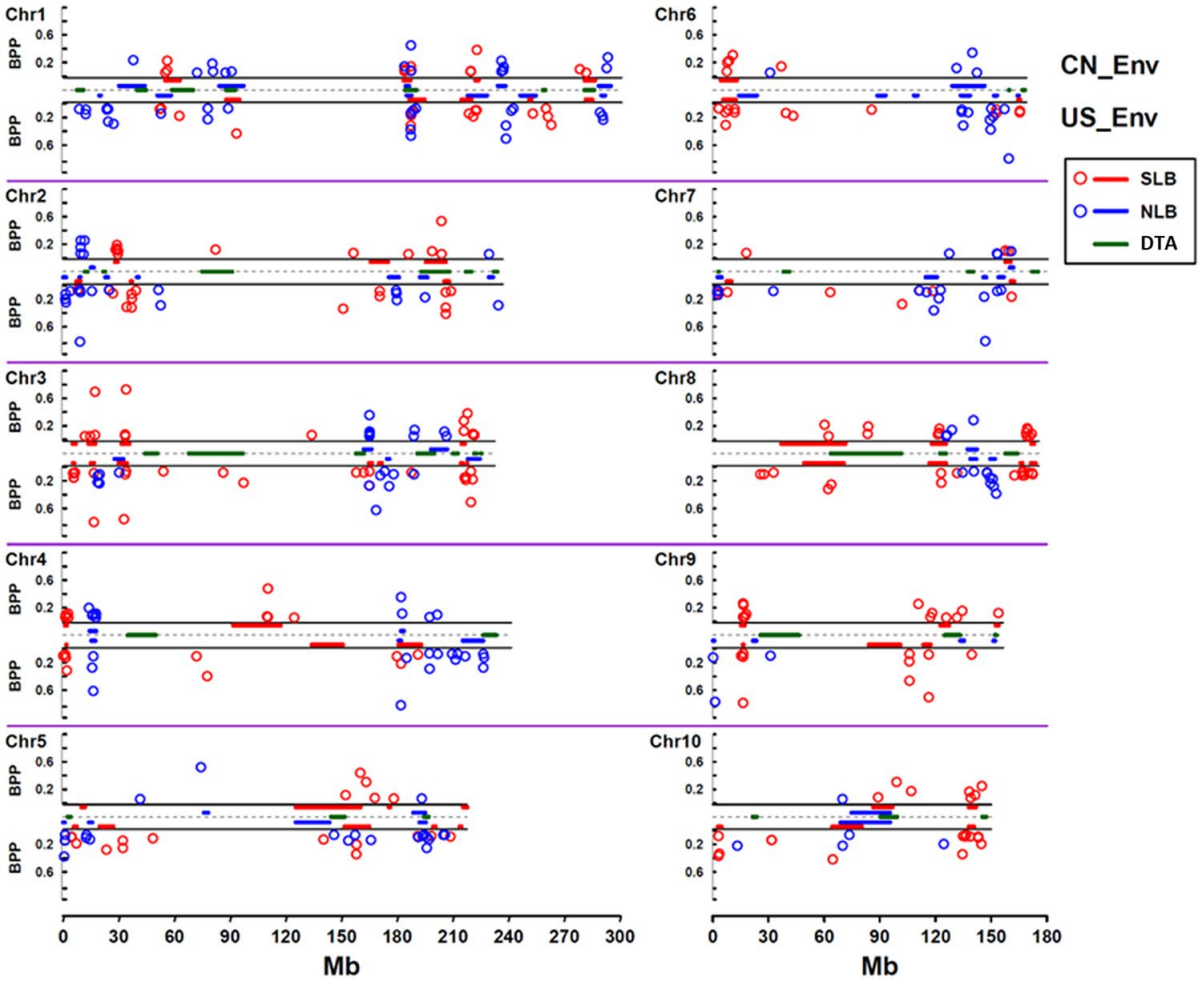
Overlap between QTLs and the identified associations. The linkage and association analyses were performed according to the different environments in the US (US_Env) and China (CN_Env). The strength of association is demonstrated as the bootstrap posterior probability (BPP). The locations of QTLs and associated loci are displayed for each chromosome, trait and panel. BPP is illustrated by the upper scale for the results in the environment of China and the lower scale for the results in the environment of the US. BPP is differentiated by colour for different traits (red for SLB and blue for NLB). QTL regions are indicated with solid lines (red for SLB and blue for NLB) at the top and bottom for the environments in China and the US, respectively. The QTL regions of DTA are indicated with green solid lines in the middle.

Integrated genotype data based on 28.5 million markers, covering the variants of maize HapMap 1, HapMap 2 and 228,212 read-depth variants (RDVs), for the 25 founder lines and B73 of the NAM population (http://www.panzea.org/) were tested for associations with SLB and NLB resistance. Using this set of genotypes, the updated NAM GWAS identified several candidate genes consistent with previous QTL fine-mapping results^31^. Subsequently, the complete marker data set was imputed for the NAM RILs using pedigree information and the 7386 reference SNPs in the genetic map. According to the residual phenotypic values obtained after accounting for QTLs on other chromosomes, a trait–marker association test was conducted by chromosome, as described by Tian *et al*.^36^. To control false positives, the analyses were repeated with partial random sampling to calculate the bootstrap posterior probability (BPP). Using the criteria of BPP > 0.05^36,37^, we detected a total of 418 marker-trait associations (in the US: 140 for SLB, 126 for NLB; in China: 96 for SLB, 56 for NLB) (Supplementary Dataset S3). Generally, most of the associated markers were clustered within or around the SLB or NLB QTL regions (Fig. 4). For SLB and NLB, 134 (57%) and 86 (47%) of the associated markers, respectively, were distributed within the QTL regions. In addition, the associated markers detected in the environments of the US and China tended to cluster in the same resistance QTL regions as the overlapping QTLs. For example, the SLB-associated markers detected in the environments of the US and China were clustered at several resistance-implicated gene-enriched SLB resistance-related unique QTLs in bins 1.06, 4.01, 6.01, and 9.02, and a similar pattern was observed for NLB-associated markers (Fig. 4). Therefore, the results from QTL mapping and GWAS were generally consistent. Moreover, although most of the resistance associated SNPs clustered within adjacent regions, exactly overlapping SNPs were rarely observed (only two SNPs for SLB, and none for NLB, were shared).

**Figure 5:**
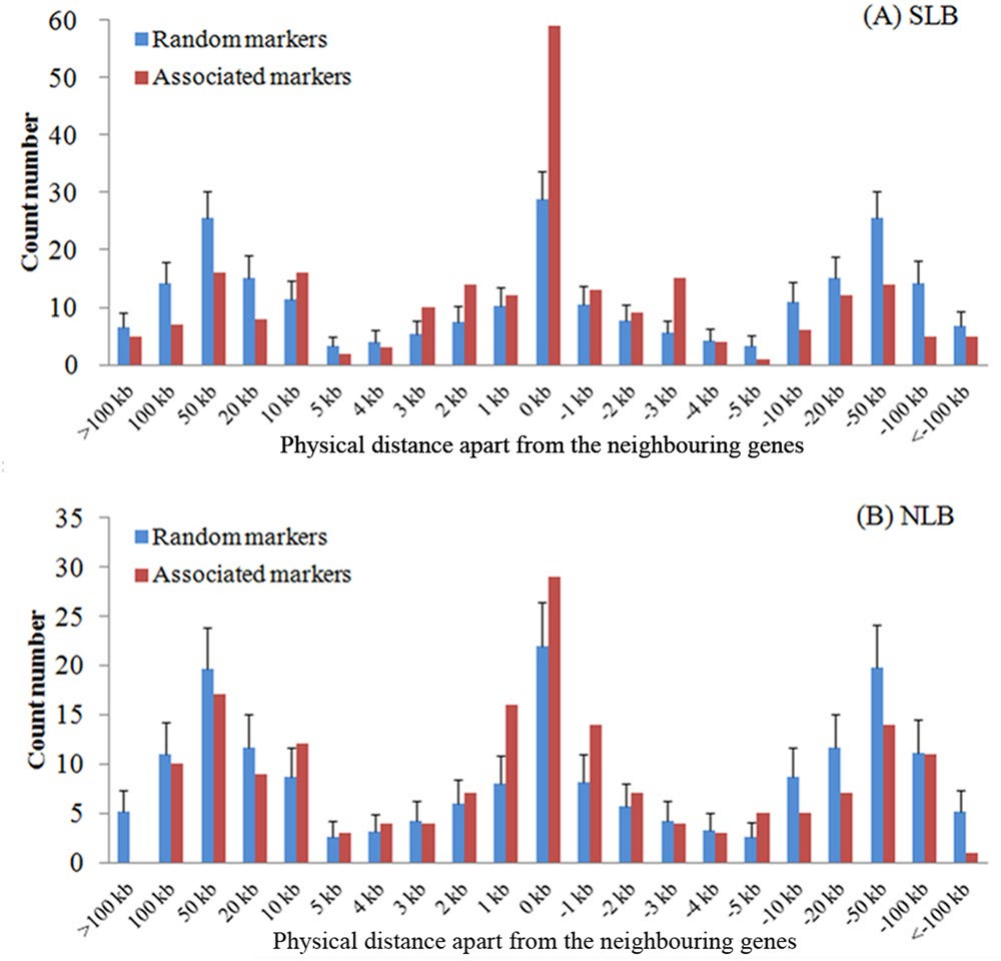
Genomic distribution test of associated markers on neighbouring genes. (**A**) SLB, and (**B**) NLB. In total, 236 SLB resistance associations and 182 NLB resistance associations were identified. The genomic distributions of the resistance associated markers on their neighbouring genes (red bars) were separately compared with a null distribution of random markers (blue bars). The null distribution was obtained by selecting an equivalent number of random markers from the applied 28.5 million marker set and calculating their proximity to different regions of their nearest genes, which was repeated 1000 times.

The genomic distribution of these resistance-associated markers (inter-gene or inner-gene regions) was compared with a null distribution (Fig. 5). The null distribution was obtained by selecting an equivalent number of random markers and calculating their potential to fall within different regions of neighbouring genes, which was repeated 1000 times. We observed a total of 59 SLB-associated and 29 NLB-associated markers within inner-gene regions, which was greater number compared with the random markers for SLB (*P* = 1.5 × 10^−7^), but not for NLB-associated markers (*P* = 8.3 × 10^−2^). We also observed that the resistance associated markers tended to be distributed within 0–2 kb of neighbouring gene regions, which covered 45% of the SLB-associated markers (*vs*. 28% for random markers, *P* = 6.5 × 10^−7^) and 40% of the NLB-associated markers (*vs*. 27% for random markers, *P* = 9.3 × 10^−4^).

### Candidate gene discovery and gene-based association analysis

According to the physical positions of the detected associated SNPs, a total of 207 and 160 genes containing or directly adjacent to associated markers (http://www.maizegdb.org/) were assumed to be potential candidate genes for SLB and NLB resistance, respectively (Supplementary Dataset S3). Among these candidate genes, 24 candidate genes for SLB and 15 candidate genes for NLB were identified based on at least two associated markers. A total of nine candidate genes (5 for SLB and 4 for NLB) were simultaneously identified using both the US and Chinese phenotypes, among which one candidate gene (GRMZM2G441903) was detected to be associated with both SLB and NLB resistance. According to the annotation analysis (http://www.maizegdb.org/), we identified 21 candidate genes that were homologous to resistance-related genes of *Arabidopsis* (http://www.arabidopsis.org/).

**Figure 6:**
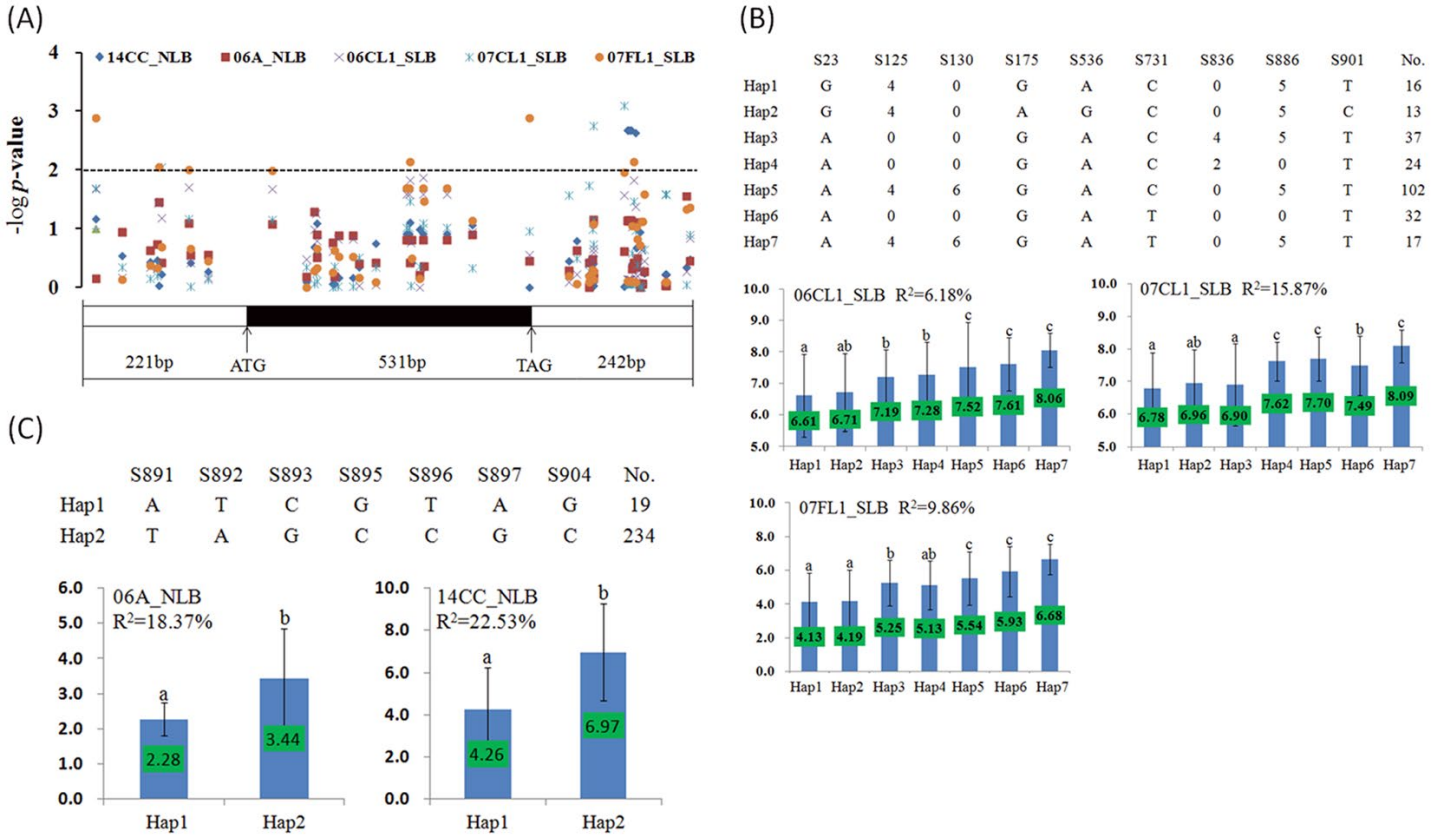
Association between polymorphisms in GRMZM2G441903 and SLB and NLB resistance (**A**) as well as the phenotypic comparisons of different haplotypes (**B**,**C**) in different environments (14CC_NLB, 06A_NLB, 06CL1_SLB, 07CL1_SLB, 07FL1_SLB). All polymorphic sites within the sequenced region with an MAF > 0.05 were used. The *y* axis represents the –log *P-value* obtained via MLM analysis for the association panel (A). Different letters indicate statistically significant differences (*P* < 0.05) according to multiple comparison analyses (**B**). According to the associated sites, the association panel was divided into seven haplotypes for SLB (**B**) and two haplotypes for NLB (**C**). The genetic contribution of haplotype was estimated using a general linear model (GLM). The averaged disease score is indicated on the middle bar for each kind of haplotype.

To obtain additional evidence of the potential roles of these genes in disease resistance, we selected four of the proposed candidate genes to conduct gene-based association analysis in a natural association population with 282 diverse maize inbred lines^38^ (http://www.panzea.org/): GRMZM2G441903 (SLB and NLB) (Fig. 6), GRMZM2G463580 (SLB) (Supplementary Fig. S3), GRMZM2G383122 (NLB) (Supplementary Fig. S4) and GRMZM2G099363 (SLB) (Supplementary Fig. S5). Significant associations were observed for the selected candidate genes except for GRMZM2G099363.

## Discussion

### A high density genetic map improves both the resolution and power of the mapping of SLB and NLB resistance QTLs

The further isolation and characterization of QRLs is an exciting frontier in QDR research^10^. Based on a linkage map including 1106 SNPs, a total of 32 SLB^26^ and 29 NLB resistance QTLs^27^ have been detected using an NAM population with 5000 RILs. With a high-density linkage map (7386 markers), improvements of both mapping power and precision were observed in the genetic analysis of the same NAM population^31^. Based on the same US phenotypes and high-density genetic maps, we identified 37 SLB and 40 NLB QTLs. Compared with the previous results^26,27^, 11 SLB and 15 NLB QTLs neither overlapped nor were adjacent to those QTLs mapped using low-density genetic maps, thus represented newly identified resistance loci. Moreover, we observed that some previously identified QTLs (5 SLB QTLs and 3 NLB QTLs) were divided into adjacent QTLs when high-density maps were used. Therefore, mapping resolution and power were both increased using the high-density genetic maps.

### Identification of more SLB and NLB resistance QTLs under diversified environments

The phenotypic analysis of QRLs has been proposed as another exciting frontier in QDR research^10^, and such analyses performed under natural conditions will be an important consideration for distinguishing minor phenotypic differences^39^. In the present study, consistent disease performance of both SLB (*R*^2^ = 0.62) and NLB (*R*^2^ = 0.56) (Fig. 1) was observed between in the US and in China. Consistent phenotypic correlations generally reveal shared plant-pathogen interactions pattern across the different environments and improve the potential for the dissection of environment-insensitive resistance QTLs. Furthermore, the different resistance reactions across the diversified environments provide an opportunity to dissect additional genetic loci, which is crucial for understanding the resistance mechanisms for these two diseases.

Combining the mapping results of the US and China, we identified a total of 49 SLB and 48 NLB resistance-related unique QTLs across the genome wide, leading to further benefits regarding the identification of resistance-related QTLs under the more diversified environments. In addition, among the 49 SLB and 48 NLB resistance-related unique QTLs detected in this study, 23 and 16, respectively, overlapped with previously identified QTLs (Supplementary Table S3). Another 20 SLB and 10 NLB QTLs have been reported in previous studies but have not been detected through NAM population. Moreover, using the phenotypes collected in China, fewer SLB and NLB resistance QTLs were detected, especially for NLB (16 QTLs in China *vs*. 40 QTLs in the US). Environmental factors, such as temperature and light, significantly affect the virulence of *Exserohilum turcicum* in maize^40^. From another perspective, this means that some disease resistance mechanisms might only be partially detected under certain environments. The information content of phenotypic data determines the obtained mapping results. In our study, the phenotypic data for NLB resistance in China were collected under only one natural inoculated environment, which might be the reason for the smaller number of detected QTLs.

### The overlap of QTLs provides valuable evidence of the shared SLB and NLB resistance mechanism

A highly positive genetic correlation between resistance to SLB and NLB has been identified^3^. According to the consensus map, several common chromosomal segments associated with resistance to multiple diseases have been identified in maize^12^. Overlapping QTLs for multiple diseases have also been evaluated in bi-parental mapping populations. However, different results have been obtained, suggesting that overlapping QTL can be detected in some studies^8,9,41–43^, but not in others^44,45^. In the present study, we observed moderate phenotypic correlations between resistance to SLB and NLB in both the US (*R*^2^ = 0.25) and China (*R*^*2*^ = 0.35). In addition, we observed that one-third of the SLB and NLB resistance regions overlapped. This overlap might be the genetic basis of the phenotypic correlations between SLB and NLB resistance, although some correlations might be caused by the potential linkage of neighbouring resistance QTLs. In our study, a region in bin 1.06 was identified to be associated with SLB and NLB resistance, and DTA. One QTL in this region was previously identified as conferring to resistance to NLB and Stewart’s wilt^46^. Additionally, this region has been identified as one of the common chromosomal segments associated with resistance to multiple diseases^12^. As such, this region is a hot-spot for candidate gene discovery of R-genes for multiple diseases.

### Co-localization of resistance QTLs and resistance-implicated genes provides clues for the identification of SLB and NLB resistance-related genes

Numerous R-genes (mainly referring to NB-LRR genes) have been identified in many plant species, and these genes have been shown to confer resistance to a wide range of pathogens^13^. R-genes, mainly referring to qualitative resistance, generally provide high levels of resistance by inducing localized cell death. However, the cell death is needed for the growth of pathogens that feed on dead tissue (necrotrophs)^47^. Therefore, there was complex relationship between the genetic base of qualitative resistance and quantitative resistance. Some QRLs have been suggested to be simply weaker forms of R-genes^23^, which is important for the genetic dissection of QDR in plants, including resistance to SLB and NLB. For example, marked changes in the expression of several NB-LRR genes have been observed after SLB infection^29^. A qualitative recessive gene for SLB, *rhm*, has been mapped to the distal end of the short arm of chromosome 6^48^. Several SLB resistance-related QTLs have also been mapped to bin 6.01^12,26,49,50^. In the present study, we observed eight resistance-implicated genes, including 6 NB-LRR genes, clustered within an SLB resistance-related unique QTL in bin 6.01 (Supplementary Fig. S2). This result might provide the direction for identifying a candidate gene for this resistance hot-spot. We also identified two SLB resistance-related unique QTLs covering more than 10 resistance-implicated genes in bins 4.01 (12 resistance-implicated genes, including 11 NB-LRR genes) and 10.01 (15 resistance-implicated genes, including 11 NB-LRR genes), although few resistant QTLs have been mapped within these two regions. Additionally, within the region related to multiple-disease resistance in bin 1.06^12,46^, we identified both SLB and NLB resistance QTLs, and two resistance-implicated genes fell precisely within the overlapping region of SLB and NLB resistance QTLs. As such, intensive co-localization of resistance QTLs and resistance-implicated genes was observed, and provided clues concerning the potential roles of these resistance-implicated genes in the molecular mechanisms of quantitative resistance, especially for NB-LRR-type genes and disease resistance homologues.

### Various genes involves in QDR against SLB and NLB

In previous studies of an NAM population using 1.6 million markers, hundreds of resistance candidates have been identified for both SLB^26^ and NLB^27^. However, only 10 SLB and 11 NLB candidate genes (Supplementary Dataset S3) were repeatedly identified using the high-density genotypes with 28.5 million markers in the same NAM population. Moreover, among these repeatedly identified candidates, one SLB (GRMZM2G099363) and two NLB (GRMZM2G441903, GRMZM2G380518) candidate resistance genes were also co-identified using the phenotypic data of both the US and China. These genes might be important candidates for resistance to SLB and NLB.

The annotations of the identified candidate genes were checked (http://www.maizegdb.org/). Similar to previous studies^26,27^, we identified several types of genes that may function in known plant disease-resistance pathways (Supplementary Dataset S3), such as genes with leucine-rich repeat (LRR) domains^51,52^, genes with serine-threonine protein kinase activity^53^, and genes involved in anti-freezing properties^54–57^. Moreover, we identified a remorin gene (GRMZM2G001973, SLB resistance related) in bin 2.06. Remorin proteins have been characterized for their roles in plant-microbe interactions. In maize, the remorin gene *ZmREM6.3* has been identified as a candidate gene of an NLB resistance QTL in bin 1.02^6^. G proteins function in the pathogenicity signalling pathways of filamentous fungi^58^. We identified six (SLB and NLB resistance related) genes involved in G-protein-coupled receptor protein signalling pathways. Moreover, among the identified candidate genes, 21 were homologous to disease resistance related genes of *Arabidopsis* involved in various resistance processes. As such, the results of the present study provide additional evidence that QDR in plants involves a range of mechanisms, including basal resistance^26,27^.

### Gene-based association analysis provides support for the possible disease resistance functions of several SLB and NLB candidate resistance genes

Several studies have demonstrated that GWAS using NAM is an effective tool for gene identification in maize^26,27,32,33,36^. However, the identification of candidate genes using GWAS alone would be arbitrary without additional evidence because the results of GWAS generally provide evidence of actual functional genes. As such, the evidence related to these candidate genes is important for understanding the mechanisms underlying quantitative resistance to necrotrophic pathogens in plants^26^. Therefore, we selected four candidate genes to conduct candidate gene-based association analysis.

The first candidate gene, GRMZM2G441903, is located in a chromosomal segment related to resistance to multiple diseases, including SLB and NLB, in bin 1.06^12,46^. This candidate gene has been identified based on six resistance associations (both SLB and NLB) with high BPP values in the environments of both the US and China. GRMZM2G441903 encodes a protein with an AN1-like zinc finger domain, that functions in DNA binding and zinc ion binding, whose homologous genes encode proteins that act as important regulators of the stress response^59,60^. Thus, it is premature to assume that this gene will likely respond to quantitative disease at this disease-resistance hot-spot. We sequenced the flanking and coding regions of this gene (Fig. 6A). In a natural association panel, significant associations were detected in the MLM Q + K model for both SLB and NLB resistance across different environments. According to the SLB resistance-associated sites (SNPs and insertion-deletions), seven types of haplotypes were identified for this gene, which were divided into three groups according their resistance performance (Fig. 6B). For NLB resistance, only two types of haplotypes were identified, and consistent performance was observed for the identified haplotypes across environments (Fig. 6C). As such, the results of gene-based association analysis have provided positive evidence for the potential role of this gene in multiple-disease resistance.

The second candidate gene, GRMZM2G463580, a representative of the LRR-type genes, represents a direct hit of two SLB-associated markers (with the highest BPP of 0.79) and encodes a member of the LRR protein kinase family. We sequenced three exons of this gene (Supplementary Fig. S3A). Significant associations for SLB-resistance were detected across all three environments (Supplementary Fig. S3B). According to the associated sites, six types of haplotypes were identified for this gene in the association panel, and notable resistance performance was observed among different haplotypes. Therefore, potential contributions of LRR genes to SLB resistance were supported by the gene-based association analysis conducted in the present study.

The third candidate gene, GRMZM2G383122, represented a direct hit of an NLB-associated marker (with a BPP of 0.81) and encodes BCL-2-associated athanogene 3 (BAG3). The homologue of this gene in *Arabidopsis* encodes a BAG protein, which regulates apoptotic-like processes ranging from pathogen attack and abiotic stress to plant development (http://www.arabidopsis.org/). This gene was proposed as a candidate gene associated with broad-spectrum resistance. We sequenced four exons of this gene (Supplementary Fig. S4A), only one significant association (insertion-deletion) was detected in one environment (Supplementary Fig. S4B). Differences in resistance performance could be observed between two haplotypes, although a significant difference was observed in only one environment. Therefore, this broad-spectrum candidate resistance gene might also play a role in quantitative resistance.

The last gene, GRMZM2G099363 (*ZmCCoAOMT2*), was identified based on four associations with SLB (with the highest BPP of 0.78) in the environments of both the US and China. Its homologue in *Arabidopsis* encodes an S-adenosyl-L-methionine-dependent methyltransferase superfamily protein (CCoAOMT1), which has been implicated in O-methyltransferase activity^61^. Recently, this gene was demonstrated to confer quantitative resistance to multiple pathogens, including SLB in maize^28^. Unfortunately, no significant associations were detected within the three sequenced exons of this gene, possibly due to the lower minor allele frequency (MAF) of resistance alleles in the association panel or the over-control in MLM Q + K model during association analysis. Another important reason is that we only sequenced exons of this gene, but the causal variation might locate in the promoter or 3′-UTR instead of coding region of the target gene, because it has been proven that the level of gene expression might be the genetic reason of its disease resistance^28^. However, the successful validation of this gene through the fine mapping approach hints the potential value of GWAS results for SLB and NLB resistance in NAM according to the more experimental conditions and the high density markers.

In summary, three candidate genes homologous to known plant resistance genes showing significant associations with disease resistance have been identified, based on which additional evidence has been obtained according to the assumption that common resistance-implicated genes, even across different plants, may provide important material for achieving QDR in maize.

## Methods

### Plant materials and phenotyping

Previously, a maize NAM population was used to investigate the genetic architecture of several complex traits, including resistance to SLB^26^ and NLB^27^. This NAM population includes 5000 RILs obtained from crosses of 25 diverse inbred lines (B97, CML52, CML69, CML103, CML228, CML247, CML277, CML322, CML333, Hp301, Il14H, Ki3, Ki11, Ky21, M37W, M162W, Mo18W, MS71, NC350, NC358, Oh43, Oh7B, P39, Tx303, and Tzi8) with one common parent, B73^25^.

The SLB disease scores for this NAM population have been reported with one replication in three environments in the US, i.e., the summers of 2006 and 2007 in Clayton, NC, and the winter of 2007 in Homestead, FL^26^. The NLB resistance of the NAM population was also scored with one replication under three artificially inoculated environments in the US, in Aurora, New York^27^.

Moreover, the disease scores of this NAM population were evaluated with one replication in naturally inoculated conditions in China in 2010 and 2011 (Supplementary Dataset S1), during which obvious differences in the resistance to SLB or NLB were observed among the families and RILs at several locations, i.e., Sanya in Hainan Province (18.15°N, 109.30°E), Tongnan in Chongqing Province (30.03°N, 106.22°E), and Xinxiang in Henan Province (35.19°N, 113.53°E) for SLB and Changchun in Jilin Province (43.90°N, 125.32°E) for NLB. The SLB or NLB disease resistance scores of each RIL were recorded 30 days after anthesis based on assessment of the number and area of lesions on the leaves above and closest to the ear^26^. The disease scores were assigned according to a nine-point scale, in which a higher score indicates greater SLB or NLB susceptibility^49^.

Additionally, a total of 488 RILs for SLB and 529 RILs for NLB, including all the RILs from the two crosses (B73 × CML322 and B73 × HP301) and other randomly selected RILs from the NAM population, were selected and artificially inoculated with the pathogen responsible for SLB (*C. heterostrophus* race O) or NLB (*E. turcicum* race 1) (Supplementary Dataset S1). All the plants in each plot were inoculated at the six- to eight-leaf stage. The artificial inoculations were conducted with two replications at Xinxiang in Henan Province (35.19°N, 113.53°E) for SLB and at Changchun in Jilin Province (43.90°N, 125.32°E) for NLB in 2013. The disease scores were recorded 30 days after anthesis using a nine-point scale standard, as described by Balint-Kurti *et al*.^49^.

As the authors’ institution is the national agricultural research organization of China (Institute of Crop Sciences, Chinese Academy of Agricultural Sciences), the authors had permission to conduct the activities described in this study, and all the experiments were supervised to comply with local regulations.

### Joint linkage analysis

Joint linkage mapping of SLB or NLB QTLs was performed using the joint stepwise regression method^35^ employing the StepwiseJointLinkagePlugin of Tassel 3.0 Standalone (https://www.maizegenetics.net/tassel). This method involves stepwise regression with the criterion of selection/removal thresholds set at *P* = 0.0001^26,27^.

### Co-localization test between resistance-implicated genes and QTLs

The co-localization between resistance-implicated genes and QTLs was evaluated by comparing the locations between the candidate genes and the QTLs detected in the NAM population. If a resistance-implicated gene fell within or overlapped with a QTL interval, then we deduced that this resistance-implicated gene co-localized with the relevant QTLs; otherwise, we assumed no co-localization. Subsequently, we calculated the actual intra-QTL ratio (proportion of co-localized resistance-implicated genes) as the number of resistance-implicated genes falling within QTL intervals divided by the total number of resistance-implicated genes. In addition, different subsets of randomly selected genes were sampled from a set of 39,423 annotated genes located across the genome (http://www.maizegdb.org/), with 1000 replications. The proportion of these randomly selected genes falling within the QTL intervals was calculated as described above and defined as the expected (by chance) intra-QTL ratio.

### Genome-wide association studies

According to the residual phenotypic values obtained after accounting for QTLs on other chromosomes, a trait–marker association test was conducted by chromosome as described by Tian *et al*.^36^. Forward regression was conducted based on the complete RIL dataset one chromosome at a time (after removing the effects of QTLs mapped on other chromosomes) to identify SNPs that were significant at *P* < 5 × 10^−8^. To control false positives, a sub-sampling-based multiple SNP model was applied^36,37^. Briefly, one hundred nonparametric bootstrap samples, sampled from 80% of each family, were analysed using stepwise regression. The bootstrap posterior probability (BPP) was subsequently calculated as the proportion of the 100 replicates in which a trait-associated marker was detected (ranging from 0 to 1). Markers detected in more than 5% of the samples (BPP > 0.05) were examined as polymorphisms in linkage disequilibrium with potential candidate genes from the B73 filtered gene set.

### Candidate gene sequencing and association analysis

The candidate gene-based association analysis was conducted in a natural association panel with 282 diverse maize inbred lines^38^. The SLB and NLB disease scores of this panel were recorded in environments in the US (http://www.panzea.org/). A total of 236 lines of this panel were also evaluated for NLB resistance under the natural conditions of Changchun in Jilin Province, China (43.90°N, 125.32°E), in 2014 (14CC_NLB) (Supplementary Dataset S1). Therefore, three sets of data on SLB resistance (06CL1_SLB, 07CL1_SLB, 07FL1_SLB) and two sets of data on NLB resistance (06A_NLB, 14CC_NLB) were obtained for the association panel.

Genomic DNA was extracted from leaf tissues using the cetyltrimethylammonium bromide (CTAB) method^62^. The genomic DNA sequences of candidate genes from the association panel were obtained via PCR amplification using primers designed from their physical positions (Supplementary Table S4). PCR was performed using high-fidelity LA Taq Mix (Takara, http://www.clontech.com/takara). The purified PCR products were cloned into pLBVector (TIANGEN, http://www.tiangen.com). The PCR products from three repetitions were directly sequenced. Initial alignment and manual refinement of the alignment were performed using BioEdit software^63^. Sites with minor allele frequencies (MAFs) >0.05 were employed for subsequent analysis. Association mapping was performed with TASSEL 2.1 using an MLM Q + K model^64,65^.

## Electronic supplementary material

Supplementary information

Dataset S1

Dataset S2

Dataset S3
