# Supplementary material for: Increased experimental conditions and marker densities identified more genetic loci associated with southern and northern leaf blight resistance in maize
Source: Sci Rep. 2018 May 1;8:6848. doi: 10.1038/s41598-018-25304-z (PMC5931595; doi:10.1038/s41598-018-25304-z)
Supplement: Supplementary file 1 — Supplementary information [file 41598_2018_25304_MOESM1_ESM.pdf]

Supplementary information:

Increased experimental conditions and marker densities identified more genetic loci associated with southern and northern leaf blight resistance in maize

Yong-xiang Li[1], Lin Chen[1], Chunhui Li[1], Peter J. Bradbury[2,3], Yun-su Shi[1], Yanchun Song[1], Dengfeng Zhang[1], Zhiwu Zhang[4], Edward S. Buckler[2,3], Yu Li[1*] and Tianyu Wang[1*]

[1]Institute of Crop Sciences, Chinese Academy of Agricultural Sciences, Beijing, China 100081

[2]Institute for Genomic Diversity, Cornell University, Ithaca, NY, USA 14853

[3]United States Department of Agriculture-Agricultural Research Service, Ithaca, NY, USA 14853

[4]Department of Crop and Soil Sciences, Washington State University, Pullman, WA, USA 99164

[*]**Correspondence:**

Y.L: liyu03@caas.cn (+86 010 62131196)

T.W: wangtianyu@caas.cn (+86 010 62186632)

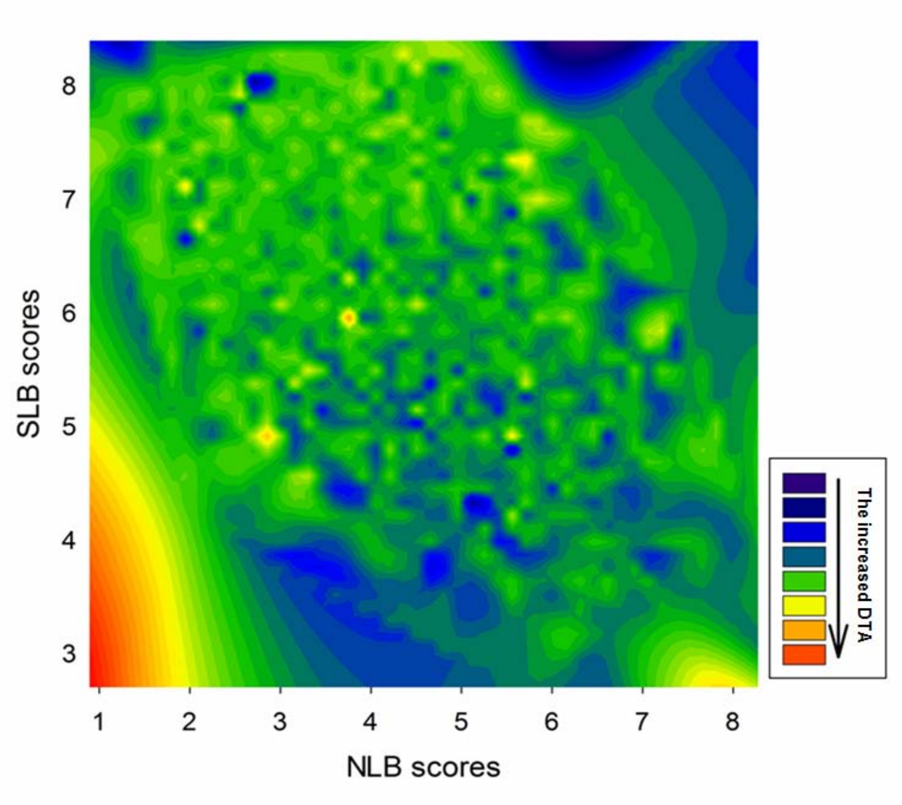

Figure S1. The contour plot for the disease scores of southern leaf blight (SLB) (*y*-axis) and northern leaf blight (NLB) (*x*-axis) and days to anthesis (DTA) (increased according to the colour ladder from dark blue to brown).

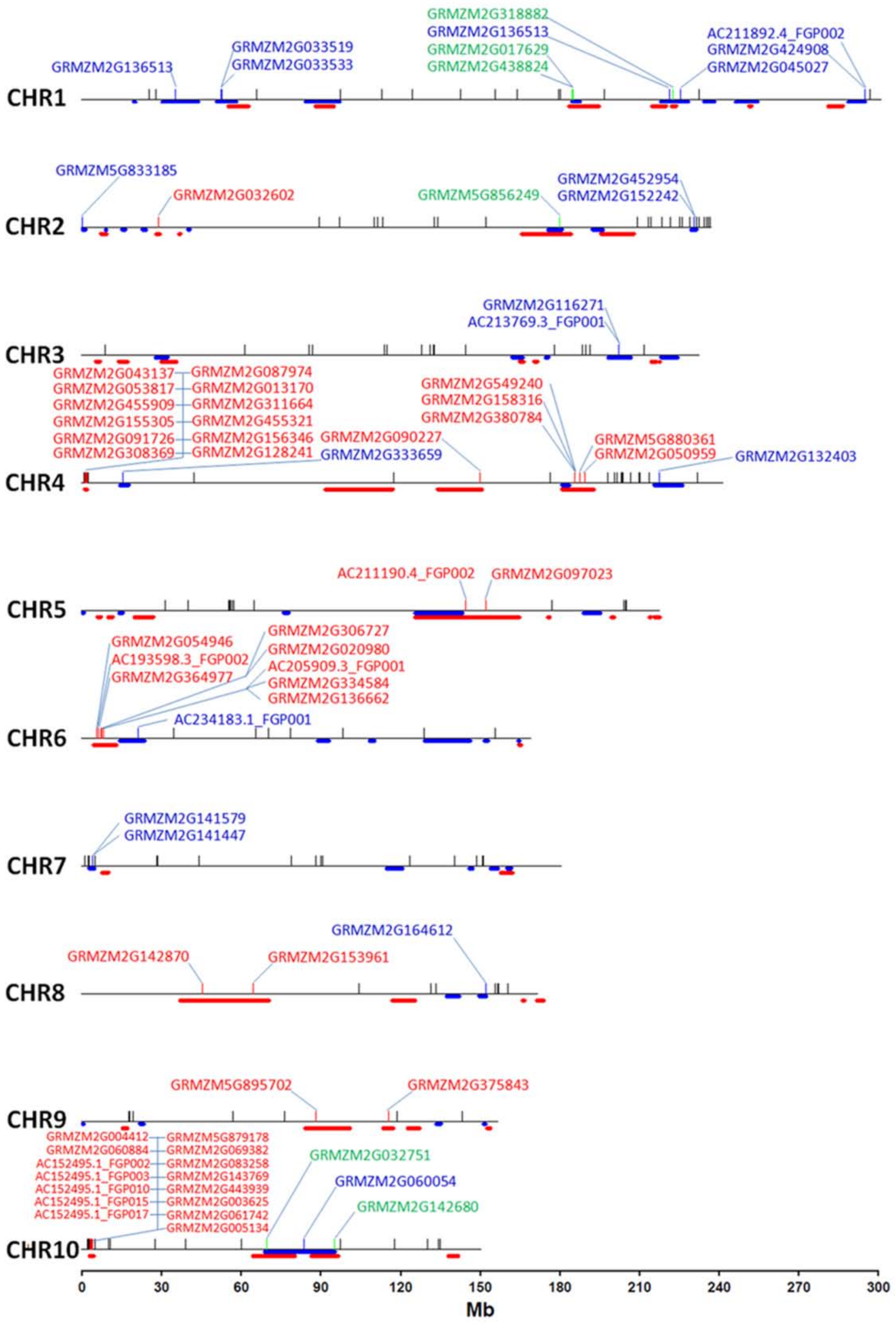

Figure S2. The distributions of resistance-implicated genes across the genome in maize. Each vertical stub across the chromosome represents the position of a resistance-implicated gene, and the SLB or NLB resistance-related unique QTLs are presented in red (SLB) or blue (NLB) solid lines below the transverse lines that represent the chromosome. Resistance-implicated genes that fell within the SLB or NLB unique QTLs are marked according to their overlapped regions (red for SLB, blue for NLB and green for the SLB and NLB shared resistance region).

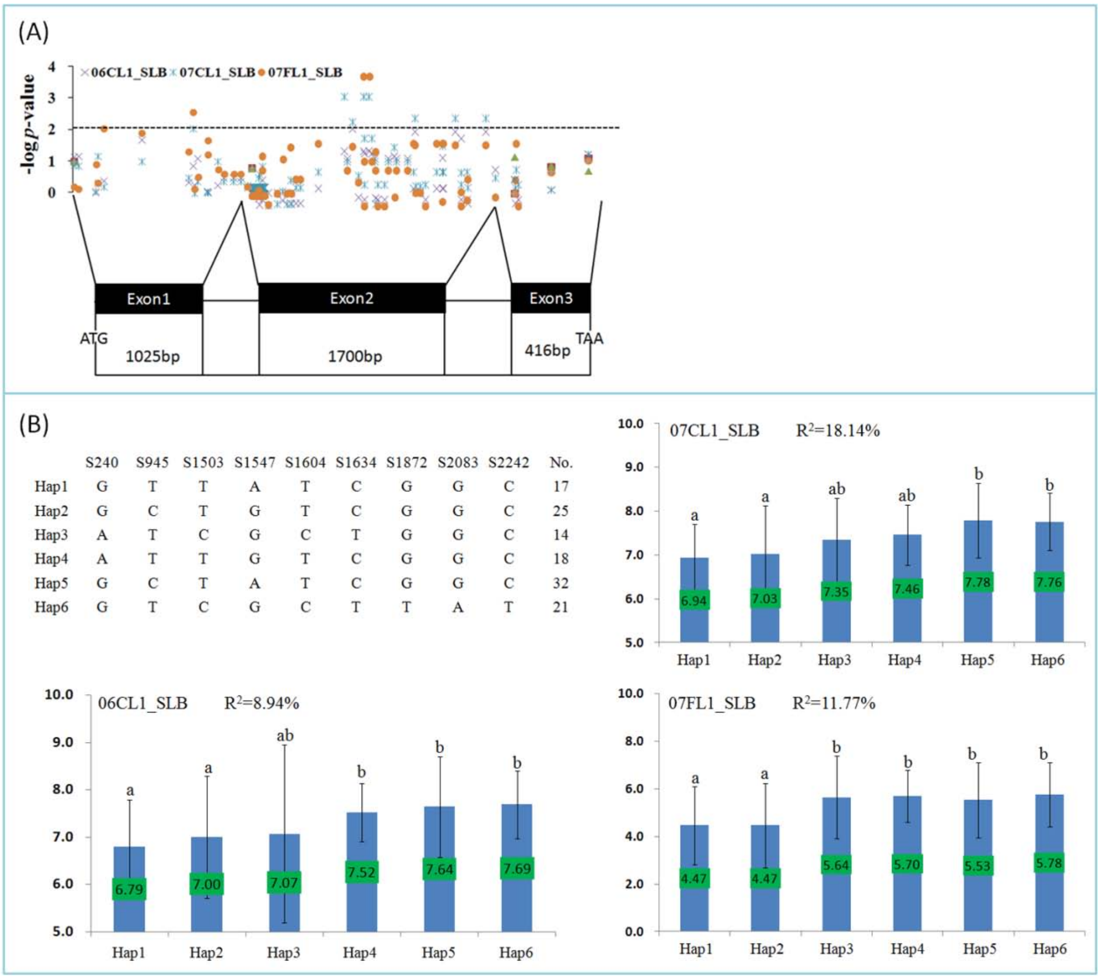

Figure S3. Association between polymorphisms in GRMZM2G463580 and SLB resistance (A) and the phenotypic comparisons of different haplotypes (B) in different environments (06CL1_SLB, 07CL1_SLB, and 07FL1_SLB). All polymorphic sites within the sequenced region with minor allelic frequency (MAF) >0.05 were used. The *y*-axis represents the –log *P-value* obtained by MLM on the association panel (A). Different letters indicate statistically significant differences ($P<0.05$) according to a multiple-comparison analysis (B). According to the nine associated sites, the association panel was divided into six haplotypes. The genetic contribution of haplotypes was estimated using a general linear model (GLM). The averaged disease score is indicated in the middle bar of each haplotype.

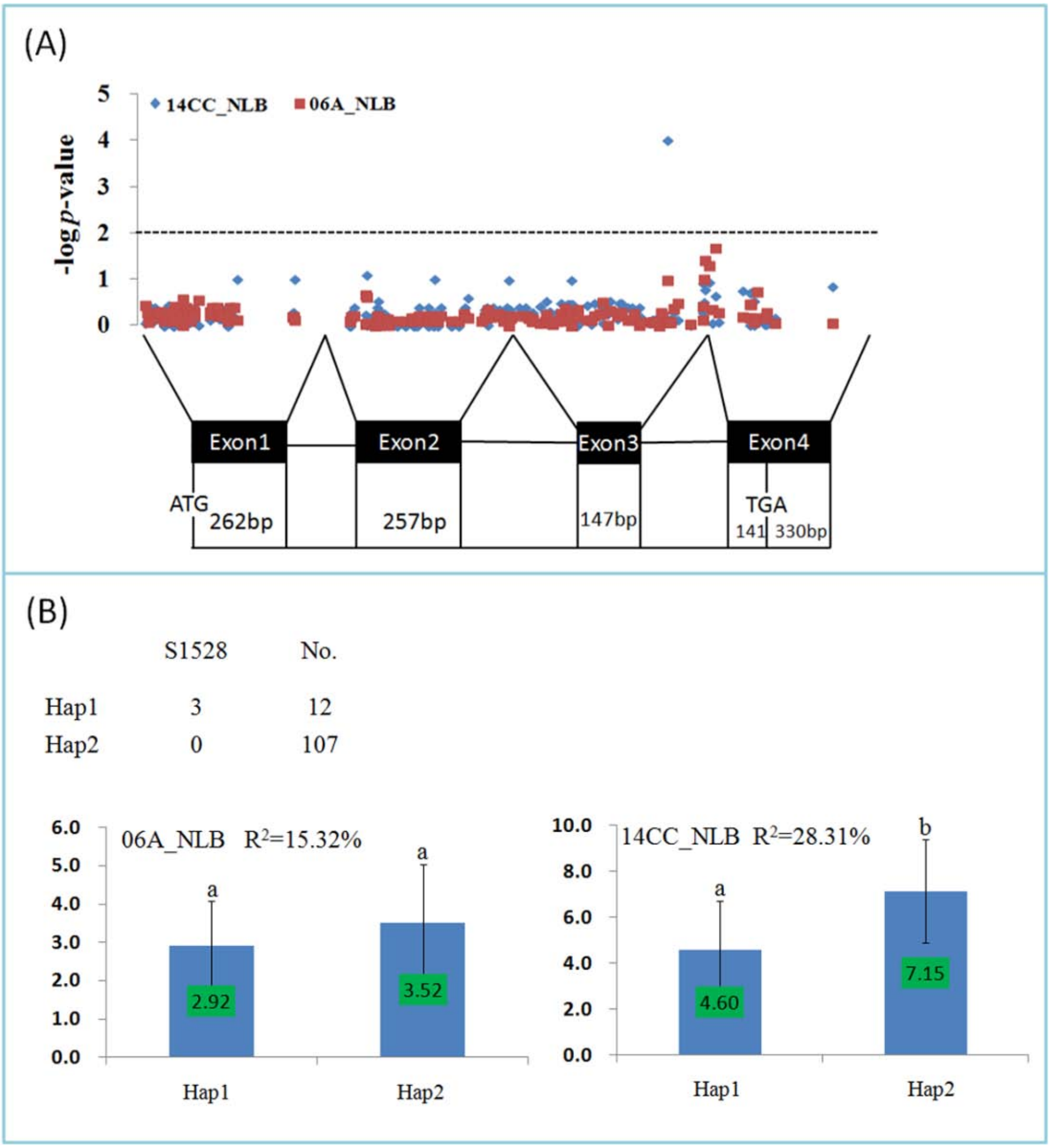

Figure S4. Association between polymorphisms in GRMZM2G383122 and NLB resistance (A) and the phenotypic comparisons of different haplotypes (B) in different environments (14CC_NLB and 06A_NLB). According to the only associated mutant, the association panel was divided into two haplotypes. The other related descriptions are presented in Figure S3.

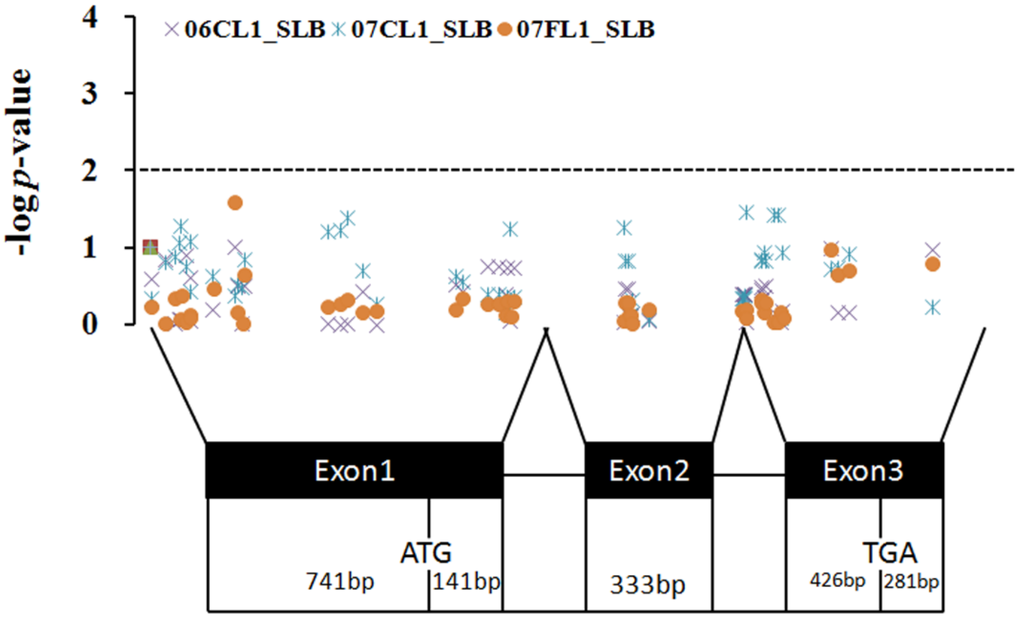

Figure S5. Association between polymorphisms in GRMZM2G099363 and SLB resistance in different environments (06CL1_SLB, 07CL1_SLB, and 07FL1_SLB). The other related descriptions are presented in Figure S3.

Table S1. Joint linkage model for DTA, SLB and NLB resistance in the environments of the United State (US) and China for the NAM population.

QTL_marker	Chr.	SS	df	MS	F	p-value	Rsq	lowerCI (AGPv2, Mb)	upperCI (AGPv2, Mb)
**BLUPs of DTA across 13 environments**									
population		140.99	24	5.87	2.87	3.69E-06	2.70E-03		
m104	1	202.16	25	8.09	3.96	1.47E-10	3.87E-03	7.51	11.01
m324	1	217.79	25	8.71	4.26	8.09E-12	4.17E-03	39.85	44.66
m398	1	125.80	25	5.03	2.46	7.16E-05	2.41E-03	58.55	70.05
m451	1	293.30	25	11.73	5.74	3.79E-18	5.61E-03	87.93	92.75
m537	1	258.42	25	10.34	5.06	3.50E-15	4.94E-03	184.37	190.34
m798	1	203.39	25	8.14	3.98	1.17E-10	3.89E-03	258.55	259.78
m915	1	145.17	25	5.81	2.84	3.23E-06	2.78E-03	280.92	286.12
m1252	2	164.21	25	6.57	3.21	1.29E-07	3.14E-03	11.65	13.15
m1337	2	140.45	25	5.62	2.75	7.00E-06	2.69E-03	21.77	22.76
m1450	2	299.99	25	12.00	5.87	1.01E-18	5.74E-03	75.01	91.12
m1588	2	135.47	25	5.42	2.65	1.56E-05	2.59E-03	192.95	207.93
m1713	2	284.60	25	11.38	5.57	2.11E-17	5.44E-03	217.03	219.86
m1821	2	161.92	25	6.48	3.17	1.92E-07	3.10E-03	232.22	233.84
m2167	3	138.72	25	5.55	2.71	9.27E-06	2.65E-03	44.22	50.75
m2177	3	161.05	25	6.44	3.15	2.23E-07	3.08E-03	67.70	115.61
m2249	3	316.12	25	12.64	6.18	4.07E-20	6.05E-03	157.80	162.21
m2403	3	189.02	25	7.56	3.70	1.61E-09	3.62E-03	190.81	199.85
m2483	3	200.09	25	8.00	3.91	2.15E-10	3.83E-03	210.08	212.60
m2572	3	173.21	25	6.93	3.39	2.70E-08	3.31E-03	221.53	222.69
m2613	3	166.29	25	6.65	3.25	9.04E-08	3.18E-03	224.97	225.37

m2966	4	329.33	25	13.17	6.44	2.89E-21	6.30E-03	34.88	49.92
m3291	4	169.31	25	6.77	3.31	5.34E-08	3.24E-03	226.25	233.43
m3484	5	130.98	25	5.24	2.56	3.19E-05	2.51E-03	2.52	4.01
m3814	5	190.92	25	7.64	3.74	1.14E-09	3.65E-03	144.43	151.64
m3967	5	167.00	25	6.68	3.27	7.98E-08	3.19E-03	194.37	196.93
m4646	6	207.21	25	8.29	4.05	5.79E-11	3.96E-03	159.58	159.75
m4772	6	145.29	25	5.81	2.84	3.17E-06	2.78E-03	166.92	168.19
m4839	7	184.28	25	7.37	3.61	3.78E-09	3.52E-03	2.96	3.26
m5031	7	134.51	25	5.38	2.63	1.82E-05	2.57E-03	38.21	41.23
m5153	7	191.13	25	7.65	3.74	1.10E-09	3.66E-03	137.56	140.29
m5413	7	163.85	25	6.55	3.21	1.38E-07	3.13E-03	172.18	175.20
m5741	8	168.38	25	6.74	3.29	6.28E-08	3.22E-03	63.61	101.48
m5808	8	636.34	25	25.45	12.45	1.05E-48	1.22E-02	122.44	125.54
m5925	8	154.81	25	6.19	3.03	6.46E-07	2.96E-03	157.62	164.15
m6434	9	229.05	25	9.16	4.48	9.73E-13	4.38E-03	25.73	46.50
m6515	9	410.46	25	16.42	8.03	2.05E-28	7.85E-03	124.99	133.39
m6735	9	193.44	25	7.74	3.78	7.24E-10	3.70E-03	151.97	152.79
m7026	10	155.00	25	6.20	3.03	6.25E-07	2.96E-03	21.48	23.73
m7063	10	416.26	25	16.65	8.14	6.25E-29	7.96E-03	90.31	99.30
m7291	10	225.07	25	9.00	4.40	2.06E-12	4.31E-03	145.29	147.26
Residual		6947.32	3398	2.04			1.33E-01		
**SLB resistance in the environment of China**									
population		33.85	24	1.41	7.72	5.47E-26	1.39E-02		
m373	1	13.71	25	0.55	3.00	8.31E-07	5.65E-03	55.07	62.86
m520	1	13.93	25	0.56	3.05	5.49E-07	5.74E-03	183.44	187.10
m673	1	13.06	25	0.52	2.86	2.75E-06	5.38E-03	222.04	223.85

m904	1	13.02	25	0.52	2.85	2.99E-06	5.36E-03	281.02	286.63
m1375	2	20.41	25	0.82	4.47	1.16E-12	8.41E-03	27.96	29.41
m1489	2	11.54	25	0.46	2.53	4.28E-05	4.75E-03	165.62	184.27
m1610	2	16.13	25	0.65	3.53	7.60E-09	6.64E-03	195.41	206.11
m1984	3	12.31	25	0.49	2.70	1.08E-05	5.07E-03	5.17	6.86
m2106	3	22.69	25	0.91	4.97	8.85E-15	9.35E-03	13.76	17.33
m2160	3	40.52	25	1.62	8.87	3.63E-32	1.67E-02	31.53	35.71
m2526	3	33.97	25	1.36	7.44	1.13E-25	1.40E-02	214.82	216.42
m2705	4	13.77	25	0.55	3.01	7.40E-07	5.67E-03	1.05	2.04
m2986	4	26.83	25	1.07	5.88	1.03E-18	1.11E-02	91.66	117.19
m3614	5	14.01	25	0.56	3.07	4.67E-07	5.77E-03	9.85	11.74
m3826	5	14.05	25	0.56	3.08	4.38E-07	5.79E-03	125.47	160.14
m3885	5	12.74	25	0.51	2.79	5.04E-06	5.25E-03	175.49	176.22
m4206	5	12.11	25	0.48	2.65	1.56E-05	4.99E-03	215.50	217.76
m4230	6	34.12	25	1.36	7.47	8.10E-26	1.41E-02	4.36	13.05
m5252	7	12.82	25	0.51	2.81	4.36E-06	5.28E-03	157.70	160.43
m5738	8	20.98	25	0.84	4.59	3.43E-13	8.64E-03	36.98	71.57
m5805	8	18.85	25	0.75	4.13	2.98E-11	7.77E-03	118.04	125.54
m6087	8	16.93	25	0.68	3.71	1.53E-09	6.97E-03	171.67	173.01
m6353	9	17.98	25	0.72	3.94	1.78E-10	7.41E-03	15.25	17.19
m6503	9	16.27	25	0.65	3.56	5.75E-09	6.70E-03	122.67	127.30
m6755	9	15.03	25	0.60	3.29	6.63E-08	6.19E-03	152.48	153.95
m7059	10	14.44	25	0.58	3.16	2.07E-07	5.95E-03	86.42	96.74
m7217	10	17.27	25	0.69	3.78	7.73E-10	7.11E-03	138.00	141.76
Residual		575.27	3149	0.18			2.37E-01		
**NLB resistance in the environment of China**									

population		535.25	24	22.30	17.93	3.40E-71	4.28E-02		
m303	1	87.67	25	3.51	2.82	3.83E-06	7.01E-03	30.22	43.89
m446	1	96.61	25	3.86	3.11	3.26E-07	7.72E-03	84.20	97.14
m524	1	101.14	25	4.05	3.25	9.04E-08	8.09E-03	184.62	186.36
m722	1	107.24	25	4.29	3.45	1.56E-08	8.57E-03	234.26	238.34
m968	1	85.70	25	3.43	2.76	6.51E-06	6.85E-03	288.61	295.02
m1286	2	153.33	25	6.13	4.93	1.19E-14	1.23E-02	15.00	16.41
m2255	3	138.82	25	5.55	4.47	1.13E-12	1.11E-02	162.00	166.15
m2443	3	88.30	25	3.53	2.84	3.23E-06	7.06E-03	198.19	206.86
m2893	4	140.34	25	5.61	4.51	7.07E-13	1.12E-02	14.28	17.82
m3172	4	132.58	25	5.30	4.26	7.83E-12	1.06E-02	182.08	183.63
m3786	5	79.22	25	3.17	2.55	3.58E-05	6.33E-03	75.94	78.03
m3949	5	83.84	25	3.35	2.70	1.07E-05	6.70E-03	188.80	195.14
m4483	6	192.26	25	7.69	6.18	4.10E-20	1.54E-02	129.09	146.38
m5269	7	97.17	25	3.89	3.13	2.78E-07	7.77E-03	160.02	161.79
m5832	8	204.91	25	8.20	6.59	6.34E-22	1.64E-02	137.31	142.23
m7046	10	93.25	25	3.73	3.00	8.31E-07	7.46E-03	74.81	95.42
Residual		4223.13	3396	1.244			3.38E-01		
**SLB resistance in the US environment**									
population		36.68	24	1.53	9.04	7.64E-32	1.08E-02		
m450*	1	27.26	25	1.09	6.45	2.72E-21	8.05E-03	87.93	94.99
m538	1	10.68	25	0.43	2.53	4.26E-05	3.16E-03	186.61	194.81
m663*	1	22.69	25	0.91	5.37	1.59E-16	6.70E-03	214.70	220.06
m759*	1	18.34	25	0.73	4.34	3.89E-12	5.42E-03	251.14	252.30
m910	1	15.21	25	0.61	3.60	4.04E-09	4.49E-03	281.66	285.20
m1179	2	15.63	25	0.63	3.70	1.62E-09	4.62E-03	7.11	9.37

m1399*	2	67.06	25	2.68	15.86	1.85E-64	1.98E-02	36.53	37.14
m1630	2	22.26	25	0.89	5.26	4.33E-16	6.58E-03	205.45	207.93
m1992*	3	20.16	25	0.81	4.77	5.86E-14	5.96E-03	5.29	5.97
m2102*	3	41.45	25	1.66	9.80	1.52E-36	1.22E-02	15.00	16.56
m2159*	3	35.71	25	1.43	8.45	2.52E-30	1.06E-02	29.87	34.28
m2265*	3	15.30	25	0.61	3.62	3.30E-09	4.52E-03	164.79	166.47
m2284*	3	18.53	25	0.74	4.38	2.49E-12	5.48E-03	170.20	171.73
m2513*	3	15.05	25	0.60	3.56	5.73E-09	4.45E-03	214.40	215.15
m2540*	3	22.72	25	0.91	5.37	1.47E-16	6.71E-03	217.52	217.77
m2707*	4	15.42	25	0.62	3.65	2.55E-09	4.56E-03	1.74	1.84
m2996	4	17.75	25	0.71	4.20	1.45E-11	5.25E-03	133.93	150.74
m3167	4	13.36	25	0.53	3.16	2.04E-07	3.95E-03	180.79	192.87
m3568*	5	12.69	25	0.51	3.00	8.18E-07	3.75E-03	5.83	7.29
m3718*	5	21.26	25	0.85	5.03	4.53E-15	6.28E-03	19.79	26.99
m3823*	5	18.84	25	0.75	4.46	1.23E-12	5.57E-03	151.64	164.88
m3985*	5	15.31	25	0.61	3.62	3.28E-09	4.52E-03	199.38	200.55
m4173*	5	12.07	25	0.48	2.86	2.83E-06	3.57E-03	213.65	214.40
m4229*	6	48.19	25	1.93	11.40	6.89E-44	1.42E-02	5.65	12.46
m4742*	6	12.08	25	0.48	2.86	2.81E-06	3.57E-03	164.55	165.56
m4947*	7	14.88	25	0.60	3.52	8.30E-09	4.40E-03	7.46	10.12
m5270	7	17.05	25	0.68	4.03	7.13E-11	5.04E-03	160.53	162.38
m5740*	8	29.37	25	1.17	6.95	1.58E-23	8.68E-03	49.24	70.51
m5794	8	14.04	25	0.56	3.32	4.93E-08	4.15E-03	116.78	125.54
m5989*	8	19.57	25	0.78	4.63	2.31E-13	5.78E-03	165.87	166.82
m6103	8	12.28	25	0.49	2.91	1.86E-06	3.63E-03	171.36	173.97
m6351*	9	38.55	25	1.54	9.12	2.12E-33	1.14E-02	16.20	16.65

m6453*	9	13.43	25	0.54	3.18	1.77E-07	3.97E-03	83.94	101.07
m6481	9	17.73	25	0.71	4.19	1.54E-11	5.24E-03	113.42	117.36
m6886	10	13.95	25	0.56	3.30	6.00E-08	4.12E-03	2.91	4.68
m7042*	10	19.24	25	0.77	4.55	4.91E-13	5.69E-03	64.45	80.25
m7177*	10	16.80	25	0.67	3.97	1.24E-10	4.96E-03	138.00	140.69
Residual		585.71	3463	0.17			1.73E-01		
**NLB resistance in the US environment**									
population		30.28	24	1.26	4.79	1.54E-13	6.92E-03		
m205*	1	26.95	25	1.08	4.09	4.23E-11	6.16E-03	19.55	20.19
m374*	1	19.15	25	0.77	2.91	1.86E-06	4.38E-03	50.84	58.15
m532*	1	73.03	25	2.92	11.09	3.31E-42	1.67E-02	185.11	187.60
m681*	1	17.03	25	0.68	2.59	2.68E-05	3.89E-03	218.08	228.41
m753*	1	28.93	25	1.16	4.39	2.44E-12	6.61E-03	246.25	254.59
m961*	1	33.73	25	1.35	5.12	1.99E-15	7.71E-03	289.98	291.93
m1067*	2	18.59	25	0.74	2.82	3.80E-06	4.25E-03	0.10	1.57
m1205*	2	36.16	25	1.45	5.49	5.16E-17	8.26E-03	8.91	9.29
m1349	2	28.57	25	1.14	4.34	4.13E-12	6.53E-03	22.76	24.24
m1410*	2	31.99	25	1.28	4.86	2.71E-14	7.31E-03	39.89	40.71
m1512*	2	19.38	25	0.78	2.94	1.38E-06	4.43E-03	175.54	180.92
m1590	2	19.78	25	0.79	3.00	8.19E-07	4.52E-03	192.28	196.30
m1783*	2	20.80	25	0.83	3.16	2.15E-07	4.75E-03	229.44	231.63
m2155	3	22.49	25	0.90	3.42	2.19E-08	5.14E-03	27.72	32.41
m2303*	3	58.02	25	2.32	8.81	7.88E-32	1.33E-02	174.58	175.80
m2598*	3	18.46	25	0.74	2.80	4.50E-06	4.22E-03	218.12	224.44
m2892*	4	36.20	25	1.45	5.50	4.85E-17	8.27E-03	15.20	17.33
m3163*	4	32.69	25	1.31	4.96	9.53E-15	7.47E-03	180.79	182.08

m3264*	4	30.55	25	1.22	4.64	2.28E-13	6.98E-03	215.46	226.25
m3450	5	28.14	25	1.13	4.27	7.71E-12	6.43E-03	0.08	0.90
m3673	5	22.04	25	0.88	3.35	4.05E-08	5.04E-03	14.03	15.58
m3807	5	30.45	25	1.22	4.62	2.63E-13	6.96E-03	125.47	143.46
m3966*	5	42.71	25	1.71	6.49	2.20E-21	9.76E-03	192.17	195.39
m4253	6	27.45	25	1.10	4.17	2.07E-11	6.27E-03	14.24	23.72
m4308	6	19.91	25	0.80	3.02	6.91E-07	4.55E-03	88.76	93.14
m4393*	6	18.21	25	0.73	2.76	6.20E-06	4.16E-03	108.29	110.28
m4493*	6	53.61	25	2.14	8.14	8.50E-29	1.23E-02	133.53	138.52
m4567*	6	32.40	25	1.30	4.92	1.47E-14	7.40E-03	151.53	153.12
m4731	6	19.58	25	0.78	2.97	1.07E-06	4.47E-03	164.28	164.89
m4837	7	18.45	25	0.74	2.80	4.55E-06	4.22E-03	2.62	4.79
m5073*	7	26.31	25	1.05	4.00	1.07E-10	6.01E-03	114.75	120.75
m5182	7	25.63	25	1.03	3.89	2.80E-10	5.86E-03	145.82	147.29
m5223	7	20.25	25	0.81	3.08	4.42E-07	4.63E-03	153.92	156.76
m5832*	8	43.68	25	1.75	6.63	4.91E-22	9.98E-03	138.79	141.91
m5872*	8	60.15	25	2.41	9.13	2.68E-33	1.37E-02	149.57	152.26
m6165	9	27.28	25	1.09	4.14	2.64E-11	6.24E-03	0.21	0.81
m6404*	9	27.27	25	1.09	4.14	2.69E-11	6.23E-03	21.77	23.44
m6531	9	23.51	25	0.94	3.57	5.41E-09	5.37E-03	133.39	135.35
m6720	9	21.81	25	0.87	3.31	5.52E-08	4.99E-03	151.26	152.08
m7038*	10	27.43	25	1.10	4.17	2.14E-11	6.27E-03	68.85	95.42
Residual		794.71	3017	0.26			1.82E-01		

Note:"*" means the QTLs overlapped with the results using the 1106 linkage map for the SLB and NLB resistance in the US environment.

Table S2. The identified SLB and NLB resistance-related unique QTLs in the environments of the US and China (CN).

Uni_QTL_Name	Chr.	Upper_Boundary (AGP v.2)	Lower_Boundary (AGP v.2)	Detected environment	Overlapped trait
UniQTL_SLB-1	1	55,071,238	62,861,084	CN	NLB, DTA
UniQTL_SLB-2	1	87,927,505	94,986,039	US	NLB, DTA
UniQTL_SLB-3	1	183,441,114	194,811,975	US, CN	NLB, DTA
UniQTL_SLB-4	1	214,696,804	220,063,596	US	NLB
UniQTL_SLB-5	1	222,044,499	223,846,758	CN	NLB
UniQTL_SLB-6	1	251,143,648	252,304,247	US	NLB
UniQTL_SLB-7	1	281,016,240	286,633,997	US, CN	DTA
UniQTL_SLB-8	2	7,113,170	9,370,056	US	NLB
UniQTL_SLB-9	2	27,964,842	29,409,244	CN	
UniQTL_SLB-10	2	36,532,760	37,143,380	US	
UniQTL_SLB-11	2	165,621,420	184,271,199	CN	NLB
UniQTL_SLB-12	2	195,411,998	207,927,887	US, CN	NLB, DTA
UniQTL_SLB-13	3	5,166,026	6,864,389	US, CN	
UniQTL_SLB-14	3	13,762,422	17,332,615	US, CN	
UniQTL_SLB-15	3	29,865,780	35,713,084	US, CN	NLB
UniQTL_SLB-16	3	164,787,824	166,467,956	US	NLB
UniQTL_SLB-17	3	170,198,345	171,730,137	US	
UniQTL_SLB-18	3	214,403,573	216,417,210	US, CN	
UniQTL_SLB-19	3	217,524,329	217,766,612	US	
UniQTL_SLB-20	4	1,054,733	2,043,040	US, CN	
UniQTL_SLB-21	4	91,656,056	117,187,022	CN	
UniQTL_SLB-22	4	133,926,556	150,743,639	US	
UniQTL_SLB-23	4	180,785,427	192,868,378	US	NLB
UniQTL_SLB-24	5	5,830,114	7,286,270	US	
UniQTL_SLB-25	5	9,846,124	11,739,901	CN	
UniQTL_SLB-26	5	19,788,296	26,985,187	US	
UniQTL_SLB-27	5	125,466,321	164,883,453	US, CN	NLB, DTA
UniQTL_SLB-28	5	175,487,002	176,219,900	CN	
UniQTL_SLB-29	5	199,380,293	200,552,003	US	
UniQTL_SLB-30	5	213,652,299	214,401,446	US	
UniQTL_SLB-31	5	215,503,134	217,759,022	CN	
UniQTL_SLB-32	6	4,361,564	13,054,242	US, CN	
UniQTL_SLB-33	6	164,552,827	165,559,706	US	NLB
UniQTL_SLB-34	7	7,463,270	10,116,910	US	
UniQTL_SLB-35	7	157,697,423	160,429,955	CN	NLB
UniQTL_SLB-36	7	160,527,310	162,377,050	US	NLB
UniQTL_SLB-37	8	36,978,572	70,512,995	US, CN	DTA
UniQTL_SLB-38	8	116,782,047	125,542,427	US, CN	DTA
UniQTL_SLB-39	8	165,865,223	166,824,598	US	
UniQTL_SLB-40	8	171,358,722	173,968,030	US, CN	NLB

UniQTL_SLB-41	9	15,246,344	17,187,897	US, CN	
UniQTL_SLB-42	9	83,939,473	101,069,822	US	
UniQTL_SLB-43	9	113,420,650	117,359,092	US	
UniQTL_SLB-44	9	122,670,173	127,302,332	CN	DTA
UniQTL_SLB-45	9	152,481,517	153,947,822	CN	DTA
UniQTL_SLB-46	10	2,914,417	4,680,884	US	
UniQTL_SLB-47	10	64,450,512	80,245,355	US	NLB
UniQTL_SLB-48	10	86,424,768	96,741,112	CN	NLB, DTA
UniQTL_SLB-49	10	137,995,210	141,761,729	US, CN	
UniQTL_NLB-1	1	19,554,169	20,192,211	US	
UniQTL_NLB-2	1	30,216,640	43,894,571	CN	DTA
UniQTL_NLB-3	1	50,843,507	58,147,064	US	SLB
UniQTL_NLB-4	1	84,195,403	97,139,837	CN	SLB, DTA
UniQTL_NLB-5	1	184,616,441	187,600,500	US, CN	SLB, DTA
UniQTL_NLB-6	1	218,082,692	228,408,957	US	SLB
UniQTL_NLB-7	1	234,257,008	238,343,143	CN	
UniQTL_NLB-8	1	246,248,916	254,589,021	US	SLB
UniQTL_NLB-9	1	288,608,985	295,018,337	US, CN	
UniQTL_NLB-10	2	95,635	1,570,730	US	
UniQTL_NLB-11	2	8,908,935	9,293,202	US	SLB
UniQTL_NLB-12	2	15,002,028	16,408,528	CN	
UniQTL_NLB-13	2	22,757,987	24,240,821	US	DTA
UniQTL_NLB-14	2	39,891,173	40,706,738	US	
UniQTL_NLB-15	2	175,544,792	180,923,801	US	SLB
UniQTL_NLB-16	2	192,283,114	196,297,682	US	SLB, DTA
UniQTL_NLB-17	2	229,437,828	231,634,193	US	
UniQTL_NLB-18	3	27,721,936	32,411,529	US	SLB
UniQTL_NLB-19	3	162,000,928	166,150,465	CN	SLB, DTA
UniQTL_NLB-20	3	174,577,580	175,804,429	US	
UniQTL_NLB-21	3	198,193,265	206,863,555	CN	DTA
UniQTL_NLB-22	3	218,120,392	224,444,931	US	DTA
UniQTL_NLB-23	4	14,284,224	17,822,823	US, CN	
UniQTL_NLB-24	4	180,785,427	183,629,572	US, CN	SLB
UniQTL_NLB-25	4	215,458,720	226,251,060	US	DTA
UniQTL_NLB-26	5	83,612	899,410	US	
UniQTL_NLB-27	5	14,032,190	15,581,585	US	
UniQTL_NLB-28	5	75,935,661	78,032,676	CN	
UniQTL_NLB-29	5	125,466,321	143,462,909	US	SLB
UniQTL_NLB-30	5	188,797,502	195,392,499	US, CN	DTA
UniQTL_NLB-31	6	14,239,607	23,722,529	US	
UniQTL_NLB-32	6	88,760,964	93,144,004	US	
UniQTL_NLB-33	6	108,288,402	110,275,761	US	
UniQTL_NLB-34	6	129,092,587	146,378,553	US, CN	
UniQTL_NLB-35	6	151,532,770	153,122,612	US	

UniQTL_NLB-36	6	164,284,303	164,891,527	US	SLB
UniQTL_NLB-37	7	2,616,676	4,789,998	US	DTA
UniQTL_NLB-38	7	114,752,286	120,750,734	US	
UniQTL_NLB-39	7	145,817,515	147,294,097	US	
UniQTL_NLB-40	7	153,922,542	156,762,939	US	
UniQTL_NLB-41	7	160,023,761	161,792,922	CN	SLB
UniQTL_NLB-42	8	137,311,246	142,228,337	US, CN	
UniQTL_NLB-43	8	149,565,696	152,263,734	US	
UniQTL_NLB-44	9	207,097	812,088	US	SLB
UniQTL_NLB-45	9	21,771,712	23,439,023	US	
UniQTL_NLB-46	9	133,386,635	135,353,570	US	DTA
UniQTL_NLB-47	9	151,257,010	152,075,617	US	DTA
UniQTL_NLB-48	10	68,852,196	95,424,687	US, CN	SLB, DTA

Table S3. The SLB and NLB resistance merged-QTLs in previous publications.

Trait	Merged-QTL*	Chr.	Upper_marker (IBM2 2008 Neighbors, coordinate)	Lower_marker (IBM2 2008 Neighbors, coordinate)	Upper_Boundary (AGP v.2, Mb)	Lower_Boundary (AGP v.2, Mb)	Overlapped environment	Reference
SLB	MSLB_1	1	4.60	52.20	0.14	5.33		[1]
SLB	MSLB_2	1	203.86	249.16	27.41	44.03		[1]
SLB	MSLB_3	1	391.02	419.82	75.76	92.98	CN	[1]
SLB	MSLB_4	1	476.23	570.21	165.41	200.41	CN, US	[1, 2, 3]
SLB	MSLB_5	1	626.95	753.55	200.72	233.56	CN, US	[1]
SLB	MSLB_6	1	782.35	847.81	240.78	259.21	US	[2, 3]
SLB	MSLB_7	1	921.05	947.87	275.38	281.65	CN, US	[1, 5]
SLB	MSLB_8	2	218.70	244.70	21.79	28.65	CN	[1]
SLB	MSLB_9	2	284.70	306.30	40.14	48.42		[1]
SLB	MSLB_10	2	342.40	411.40	64.66	186.89	CN	[2, 5]
SLB	MSLB_11	2	447.27	450.99	195.74	196.53	CN, US	[4]
SLB	MSLB_12	2	482.20	522.40	204.33	211.35	CN, US	[1]
SLB	MSLB_13	2	599.13	630.00	224.34	229.48		[3]
SLB	MSLB_14	2	650.10	702.50	232.58	235.38		[4]
SLB	MSLB_15	3	162.48	168.62	15.06	17.29	CN, US	[1, 4, 5]
SLB	MSLB_16	3	200.12	270.06	31.10	86.84	CN, US	[1, 2, 3, 4]
SLB	MSLB_17	3	287.24	328.04	114.78	148.98		[2, 4, 5]
SLB	MSLB_18	3	450.26	490.08	179.99	187.05		[1, 2]
SLB	MSLB_19	3	608.17	760.90	210.42	221.69	CN, US	[1, 2]
SLB	MSLB_20	4	101.10	140.90	5.50	11.92		[1]
SLB	MSLB_21	4	295.20	302.50	90.20	135.31	CN, US	[1]

SLB	MSLB_22	4	397.40	420.60	167.02	173.93		[1]
SLB	MSLB_23	4	565.40	579.80	202.04	217.52		[1]
SLB	MSLB_24	5	20.80	37.60	0.89	2.13		[1]
SLB	MSLB_25	5	216.30	323.10	19.68	138.16	CN, US	[2]
SLB	MSLB_26	5	323.10	368.40	138.16	168.65	CN, US	[2]
SLB	MSLB_27	5	400.00	493.70	174.41	197.59	CN	[5]
SLB	MSLB_28	5	609.40	675.44	201.99	205.55		[4]
SLB	MSLB_29	6	66.40	73.30	8.68	108.20	CN, US	[1, 4, 5]
SLB	MSLB_30	6	320.70	391.40	146.12	154.49		[2]
SLB	MSLB_31	6	391.40	410.18	154.49	156.54		[2]
SLB	MSLB_32	7	330.60	361.90	133.87	143.40		[1]
SLB	MSLB_33	7	400.00	463.96	154.18	162.15	CN, US	[5]
SLB	MSLB_34	8	100.00	136.84	8.88	14.09		[3]
SLB	MSLB_35	8	160.80	175.90	18.21	21.85		[1]
SLB	MSLB_36	8	174.12	316.20	20.70	133.56	CN, US	[2, 4]
SLB	MSLB_37	8	363.40	381.70	131.68	138.79		[1]
SLB	MSLB_38	9	66.12	228.30	9.82	83.84	CN, US	[1, 2, 5]
SLB	MSLB_39	9	266.00	340.38	105.56	131.17	CN, US	[2]
SLB	MSLB_40	9	577.20	635.20	151.15	155.10	CN	[1]
SLB	MSLB_41	10	185.10	240.21	58.00	92.91	CN, US	[5]
SLB	MSLB_42	10	332.10	335.50	130.97	132.22		[3]
SLB	MSLB_43	10	344.80	366.30	133.22	136.95		[1]
NLB	MNLB_1	1	147.46	170.00	16.37	23.68	US	[3]
NLB	MNLB_2	1	500.00	548.40	177.03	191.09	CN, US	[3, 8, 9]
NLB	MNLB_3	1	664.17	670.20	209.79	213.23		[6]
NLB	MNLB_4	1	697.10	748.50	217.95	232.74	US	[8]

NLB	MNLB_5	2	3.80	27.40	0.95	2.83	US	[9]
NLB	MNLB_6	2	105.45	216.96	9.54	21.79	CN	[3, 6, 7]
NLB	MNLB_7	2	284.70	389.25	40.59	177.95	US	[6]
NLB	MNLB_8	3	309.50	318.20	135.17	146.53		[9]
NLB	MNLB_9	3	423.29	460.00	176.55	183.12		[6]
NLB	MNLB_10	3	452.70	535.82	181.08	205.30	CN	[6, 7]
NLB	MNLB_11	3	702.20	732.70	216.79	219.64	US	[9]
NLB	MNLB_12	4	437.50	455.90	178.50	181.43	CN, US	[9]
NLB	MNLB_13	5	69.50	163.40	3.15	20.77	US	[7, 8]
NLB	MNLB_14	5	280.00	324.90	64.29	86.99	CN	[6]
NLB	MNLB_15	5	387.00	500.00	171.12	199.79	CN, US	[6]
NLB	MNLB_16	5	518.40	550.00	202.81	205.55		[6, 8]
NLB	MNLB_17	6	248.75	420.00	124.15	158.40	CN, US	[6, 3, 8]
NLB	MNLB_18	7	288.90	309.90	130.23	132.52		[6]
NLB	MNLB_19	7	309.90	360.00	132.52	149.86	US	[7]
NLB	MNLB_20	8	126.73	299.90	10.87	107.02		[3, 8]
NLB	MNLB_21	8	298.56	361.77	105.57	132.88		[6, 3]
NLB	MNLB_22	8	386.80	459.20	142.15	165.69	CN, US	[6, 8]
NLB	MNLB_23	9	1.00	64.70	2.17	9.45		[6]
NLB	MNLB_24	9	66.12	154.22	9.82	18.33		[6]
NLB	MNLB_25	9	364.49	480.19	130.92	146.08	US	[6]
NLB	MNLB_26	10	300.00	320.29	127.58	130.96		[6]

"*":

From a literature search, the SLB and NLB resistance QTLs from previous publications were checked.

Then, each merged-QTL was defined by the outmost markers of those overlapped resistance QTL in different publications.

After that, the approximate physical positions for boundary markers of each merged- QTL in AGP v.2 were estimated according to the relative positions in the map of IBM2 2008 Neighbors.

Reference:

1. Balintkurti PJ, et al. (2007). Precise mapping of quantitative trait loci for resistance to southern leaf blight, caused by *Cochliobolus heterostrophus* race o, and flowering time using advanced intercross maize lines. Genetics 176(1):645-57.

2. Balintkurti PJ, et al. (2008). Identification of quantitative trait loci for resistance to southern leaf blight and days to anthesis in a maize recombinant inbred line population. Phytopathology 98(3):315-20.

3. Zwonitzer JC, et al. (2010). Mapping resistance quantitative trait loci for three foliar diseases in a maize recombinant inbred line population-evidence for multiple disease resistance? Phytopathology 100(1):72-79.

4. Balint-Kurti PJ, et al. (2008).Identification of quantitative trait loci for resistance to southern leaf blight and days to anthesis in two maize recombinant inbred line populations. Phytopathology 98(3):315-20.

5. Zwonitzer JC, et al. (2009). Use of selection with recurrent backcrossing and QTL mapping to identify loci contributing to southern leaf blight resistance in a highly resistant maize line. Theoretical and Applied Genetics 118(5):911-925.

6. Welz HG & Geiger HH. (2000). Genes for resistance to northern corn leaf blight in diverse maize populations. Plant Breeding 119(1):1-14.

7. Dingerdissen AL, et al. (1996). Interval mapping of genes for quantitative resistance of maize to *Setosphaeria turcica*, cause of northern leaf blight, in a tropical environment. Molecular Breeding 2(2):143-156.

8. Chung CL, et al. (2011). Targeted discovery of quantitative trait loci for resistance to northern leaf blight and other diseases of maize. Theoretical and Applied Genetics 123(2):307-326.

9. Peterj BK, et al. (2010). Use of a maize advanced intercross line for mapping of QTL for northern leaf blight resistance and multiple disease resistance. Crop Science 50(2):458-466.

Table S4. The primes used for candidate gene sequencing.

Gene_ID	Primer_name	Forward primer sequence(5'-3')	Reverse primer sequence(5'-3')
GRMZM2G441903	Primer1	CCGTCCCAACGCCGTACCCA	ACAGCATGGCGATCAACACATAACC
	Primer2	GCTCTCACACGCCTCGCCTAGT	TTCCACAGCGTGTTGTCGTAGC
GRMZM2G099363	Primer3	TGAGGTAGTTGTCCTTGTCGG	CAATCCAGCAATACCCGACG
	Primer4	ATTCACCGTGCTGCCTGACTTAC	AGCCGAATGTACCATGCCTTGC
	Primer5	GCCATCACGGGAGCATAGATTACGC	CCTACGCCAGAGAGACCAAATCCAA
	Primer6	GCTGGTCAAGCTTTATTAATTTTGA	AGGTTGTTGGTGGCCGGGTT
	Primer7	ATCTATGCTCCCGTGATGGC	GGTTGACACTGCTGGTCGTT
	Primer8	GAACCGTGGAAAGTTCCTGC	TCTTTGTGCTAGCTGGCTGT
	Primer9	ACGACGATTCTTCCCCCTTG	ACCTTCTTGTCGCGCTGAG
	Primer10	TCATGTGGGTTGACACTGCT	CGAATGTACCATGCCTTGCC
	Primer11	GAGGGTCAACACCAAACGC	CACGCGGCCTCGGATTTT
	Primer12	TTTGACGGGGTACGCTTGC	CACGCGGCCTCGGATTTTG
GRMZM2G463580	Primer13	AGTATTTTAGCGGACGGTTCGAGTG	GTTCTGGCTCCTCACCCATTAATTC
	Primer14	TATATCAAACGAGAAGCTTCTGCAG	AGCAAGTTATTAGACGCTTCAAAAA
	Primer15	GTTCCAGCTCTAATGTGAAGCTGTA	TATGCTCTTTTTGAATTGTGATACC
	Primer16	TTTAAAGAAGCTTAACTCCTGGTCT	GGAAAAGTGATTGGTGTTCTGTA
	Primer17	GCAAACAAATTCACTTGTCACTGAT	CATTGTGGCTTAGGTCCAGGTTA
	Primer18	AGCAAGTYATTAGNCGCTTCARAAA	TCCGTGGGAATAAGCAGAACC
	Primer19	GCTGGGAGGGGGTGAGATG	TAAGACCGATGTTGGTGCTGATAGT
	Primer20	CAGCACCAACATCGGTCTTACT	TGTATCGGCTATCTCCCATATTCTC
	Primer21	AATCACTGTCAGCCACCAATC	TGAGGACACTCTGGACCTTATCT
	Primer22	GGTTGAATTAGGCCTGTACAATACC	TCACCATACTCTGTTTGTGCCA
GRMZM2G383122	Primer23	TCATGTTCATATGCAGGGACAGT	GATTTTGTCTGGTTCAGTCAGTGTG
	Primer24	CGGGTGACAAATATTCGAGAACAGA	TTCCCCTGATACGGAGTTTTGAGTC
	Primer25	GAGCCGAGTGGCTAAATGGA	AGGGAGCGATTCGTTGGTTT
	Primer26	GCCCACCCATAGGCAGATAC	GGGGGAAGATAGCGACACG
	Primer27	GGCCCACCCATAGGCAGATA	CCTTGGAGTGCGGGAAGC
	Primer28	ACCAACGAATCGCTCCCTTT	GGCAGGCAGGTTGTTAATGG
	Primer29	ACCAACGAATCGCTCCCTTT	AGGAGACGCACAGCTTTCAG
	Primer30	CGCACCCCGTGTGAAATGT	CATTCAATCGCACCTCCCATG
	Primer31	TGCTGCTTCTCTTAATTATGCCGAC	ATTCAGTTGGCAGCATGCGC
	Primer32	CAAGATATAGGATGCAAAAGTCCGT	AGAACGGTGTCGCTTTCCTTTAT
	Primer33	CAACGCTTTAGGGGGTGTTTG	CGTGCCCAGCATAAGCATCA
	Primer34	GCAAGCCAACGCTTTAGGG	GTGCCCAGCATAAGCATCAT
